# Shielding Surfaces from Viruses and Bacteria with a Multiscale Coating

**DOI:** 10.1002/advs.202201415

**Published:** 2022-06-03

**Authors:** Deepu Ashok, Mahdiar Taheri, Puneet Garg, Daryl Webb, Pawan Parajuli, Yi Wang, Bronte Funnell, Bradley Taylor, David C. Tscharke, Takuya Tsuzuki, Naresh K. Verma, Antonio Tricoli, David R. Nisbet

**Affiliations:** ^1^ Laboratory of Advanced Biomaterials Research School of Chemistry and the John Curtin School of Medical Research The Australian National University Canberra 2601 Australia; ^2^ Nanotechnology Research Laboratory Research School of Chemistry The Australian National University Canberra 2601 Australia; ^3^ Laboratory of Advanced Nanomaterials for Sustainability Research School of Electrical Energy and Materials Engineering The Australian National University Canberra 2601 Australia; ^4^ Centre for Advanced Microscopy Australian National University Canberra 2601 Australia; ^5^ Division of Biomedical Science and Biochemistry Research School of Biology The Australian National University Canberra 2601 Australia; ^6^ John Curtin School of Medical Research Australian National University 131 Garran Road Acton ACT 2601 Australia; ^7^ Nanotechnology Research Laboratory Faculty of Engineering The University of Sydney Sydney 2006 Australia; ^8^ The Graeme Clark Institute Faculty of Engineering and Information Technology and Faculty of Medicine Dentistry and Health Services The University of Melbourne Melbourne 3010 Australia

**Keywords:** antibacterial, antiviral, multiscale coatings, infections, ZIF‐8 metal–organic frameworks (MOFs)

## Abstract

The spread of viral and bacterial pathogens mediated by contact with surfaces is a leading cause of infection worldwide. COVID‐19 and the continuous rise of deaths associated with antibiotic‐resistant bacteria highlight the need to impede surface‐mediated transmission. A sprayable coating with an intrinsic ability to resist the uptake of bacteria and viruses from surfaces and droplets, such as those generated by sneezing or coughing, is reported. The coating also provides an effective microbicidal functionality against bacteria, providing a dual barrier against pathogen uptake and transmission. This antimicrobial functionality is fully preserved following scratching and other induced damage to its surface or 9 days of submersion in a highly concentrated suspension of bacteria. The coatings also register an 11‐fold decrease in viral contamination compared to the noncoated surfaces.

## Graphical abstract

The development of a ZIF‐8 metal–organic framework‐hierarchical dual functional coating with durable properties that can resist pathogen adhesion is reported. The coating is mechanistically characterized for both antiviral and antibacterial properties, registering excellent performance in reducing the surface residence time of viruses and resisting formation of biofilms by bacteria.

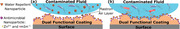

## 1 Introduction

COVID‐19 has highlighted the global risk associated with the transmission of pathogens, and its rapid and severe impact on human health and the global economy.^[^ ^1^, ^2^ ^]^ There is broad evidence that surfaces facilitate pathogen dissemination, leading to widespread transmission throughout the population.^[^ ^3^ ^]^ Surface contamination also plays a major role in the evolution of antibiotic‐resistant bacterial strains.^[^ ^4^ ^]^ Alternatives to traditional surface disinfection approaches are necessary as these are presenting declining efficacy and are often laborious and ineffective as recontamination is triggered episodically.^[^ ^5^ ^]^ Alternative approaches to prevent bacterial contamination of surfaces rely on the use of bactericidal coating materials including, silver, copper, zinc, and titanium dioxide along with other inorganic nanoparticles and quantum dots.^[^ ^6^, ^7^ ^]^ Among these, silver nanoparticles are the most widely used bactericidal agent. Recent reports highlight their limitations, including the significant variations in potency between microbial species and harmful side effects due to the accumulation of high concentrations of silver ions within the tissue.^[^ ^8^ ^]^ Furthermore, bactericidal agents fail at protecting surfaces from contamination by viral pathogens.

Recently, the use of water repellent coatings that can be deployed on high touch surfaces, such as hospital infrastructure, has shown promising efficacy in preventing bacterial and viral contamination.^[^ ^6^, ^9^ ^]^ Bacterial contamination of surfaces starts with the adhesion of a few planktonic cells leading to biofilm formation.^[^ ^10^ ^]^ Viruses can spread indirectly through contact with contaminated surfaces (fomites) and this could lead to multiple infections within the community through a wide range of environments like hospitals, nursing homes, schools, and restaurants.^[^ ^11^, ^12^ ^]^ Nonenveloped viruses (e.g., reoviruses) are known to reside on surfaces for several weeks causing large outbreaks in the healthcare facilities.^[^ ^11^ ^]^ Enveloped viruses (e.g., coronaviruses) are found to persist on inanimate surfaces and remain infectious for up to a week.^[^ ^2^, ^13^ ^]^ The engineering of water repellent surfaces can prevent this surface‐persistence of pathogens by the formation of an air layer between the contaminated liquid and solid surface. This provides a steep mechanistic barrier to biofilm formation or viral‐persistence.^[^ ^14^ ^]^

Inspired by these properties, recent studies (Table S1, Supporting Information) have begun to investigate bacterial colonization of water repellent surfaces.^[^ ^15^ ^]^ While an initial reduction of bacterial growth was observed on such surfaces, to date there is a lack of direct evidence on the mechanism preventing bacterial adhesion and growth. Furthermore, the observed bacteria inhibition is short‐lived with bacteria colony densities converging toward those on nonwater repellent surfaces within 4–6 h of immersion in a bacterial suspension. Water repellent coatings also often provide insufficient robustness for real‐world applications, due to the intrinsic fragility of the nanoscale roughness required for attaining a Cassie–Baxter water repellent state.^[^ ^16^ ^]^

Here, we report a multifunctional coating able to shield surfaces from both viral and bacterial pathogens. Initially, we engineered a water repellent coating consisting of a sprayable microscale polymer component made of interpenetrating polymer network (IPN) of polyurethane (PU) and polymethyl methacrylate (PMMA), on top of which superhydrophobic nanoparticles of fluoro functionalized silica (FS) is sprayed. PU and PMMA are synthesized in juxtaposition and their polymer chains interpenetrate each other at the molecular scale to form the IPN suspension (Figure S1, Supporting Information).^[^ ^17^ ^]^ IPN accommodates hybrid tough characteristics of PMMA and the rubbery nature of PU and when sprayed, it forms an elastically deformable film with a microtextured surface. The role of IPN here is to act as a gluey interface between the substrate and the FS nanoparticles, resulting in high abrasion resistance of the overall coating structure. FS, synthesized by functionalizing silica nanomaterials with fluorine‐precursors is a highly performing water‐repellent nanomaterial offering water contact angles above 150° and static water contact angles of 0° (Figure S2, Supporting Information).^[^ ^16^, ^18^ ^]^ Hence the IPN‐FS water repellent coating of ours features a micro–nano textured hierarchical roughness with lotus leaf‐like characteristics that maintain superhydrophobic properties up to 100 cycles of abrasion under load. Our water repellent coating structure can provide 99.94% and 99.85% initial reduction in both Gram‐positive and Gram‐negative bacteria when submerged in a contaminated liquid. Using this coating, we gained the first direct insights on the ability of these surfaces to prevent bacterial adhesion and on the eventual failure of this shielding mechanism, amid prolonged immersion in contaminated liquids or due to physical damage to the coatings. Using microscopy, we illustrated how the bacterial cell–surface interactions could give cues to understanding biofilm formation and how this knowledge could be exploited to develop anti‐biofilm coatings. We use these learnings to engineer a coating that provides enhanced and prolonged resistance to pathogen contamination via a dual surface shielding mechanism (called a dual functional coating).

**Figure 1:**
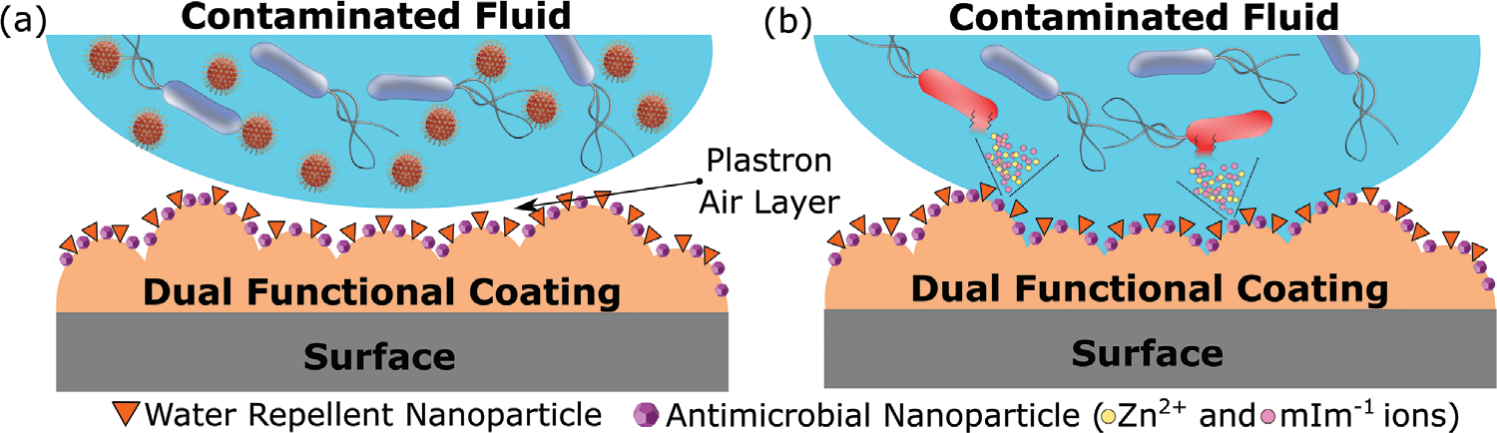
Dual functional coating for shielding surfaces from bacteria and viruses. a) Schematic of the dual‐functional coating with the air layer or plastron preventing surface contamination by pathogens contained in a liquid environment. b) Schematic of the localized loss of superhydrophobicity of the dual‐functional coating and antimicrobial effect of ZIF‐8 nanoparticles.

Previous studies have reported dual‐mechanistic repellent antibacterial technologies on fabrics characterized by multilayers on cotton fabric. The layers are usually composed of a reservoir of biocidal material on which functionalized nanorough material (usually funtionalized silane) is coated to offer repellent characteristics (Table S1, Supporting Information).^[^ ^19^ ^]^ These studies were specific in terms of their application and did not comment on its applicability in other environments. Few other studies developed dual‐mechanistic coatings for wider applications including clinical and marine environments (Table S1, Supporting Information).^[^ ^20^ ^]^ Compared to these studies, we went multiple steps ahead by demonstrating the mechanism and its collapse, along with characterizing the coating in different conditions in an effort to simulate different environment of applications. We also extended the applicability of our coating in preventing viral persistence on surfaces as well. Our dual‐functional coating consists of hydrophobic nanoparticles of zeolitic imidazolate frameworks (ZIF‐8) mixed with FS, sprayed on top of the IPN layer, essentially becoming an improvement to the initial water repellent surface (**Figure** **1**a). Dual‐functional coating boasts superhydrophobic properties whilst also accommodating bactericidal zinc ion reserves in the form of ZIF‐8. ZIF‐8 is a zinc‐based metal–organic framework (MOF), resembling the zeolite topology, with 2‐methylimidazole (HmIm) as the linker.^[^ ^21^ ^]^ ZIF‐8 is gaining a lot of attention recently because of its hybrid framework and flexibility it offers in a surface modification that helps in the rational tuning of surface properties.^[^ ^22^ ^]^ ZIF‐8 also boasts better thermal and chemical properties compared to other MOFs, paving its way into biomedical applications in tissue engineering, drug delivery and antimicrobial therapy.^[^ ^22^, ^23^ ^]^ Hence, our dual‐functional coating features both enhanced and robust water repellence, based on a multiscale roughness, and a highly effective localized microbicidal action triggered by a stimuli‐responsive ion release mechanism (Figure 1). The secondary mechanism helps the coating retains its antimicrobial performance following either significant physical damage or extended submersion in a bacterial suspension for more than 9 days (Figure 1b). This is 22 times more than the best performing water repellent coating reported. Our dual‐functional coating also registers an immediate 11‐fold decrease in viral pathogen contamination. Our coating shielding mechanism is effective in both dry (Figure 1a) and wet environments (Figure 1b), providing means to inhibit pathogen contamination of hospital infrastructure, air and water filters, ship hulls and other surfaces at a high risk of mediating pathogen transmission. Our coating is engineered through a simple and scalable technique with a careful choice of materials that resulted in its ultra durability. We also believe that our unique characterizations reported on understanding the mechanism behind the antimicrobial and antiviral effects could significantly advance the research in the area of antipathogen technologies.

## 2 Results and Discussion

### 2.1 Engineering a Water Repellent Coating for Shielding Surfaces from Bacteria

**Figure 2:**
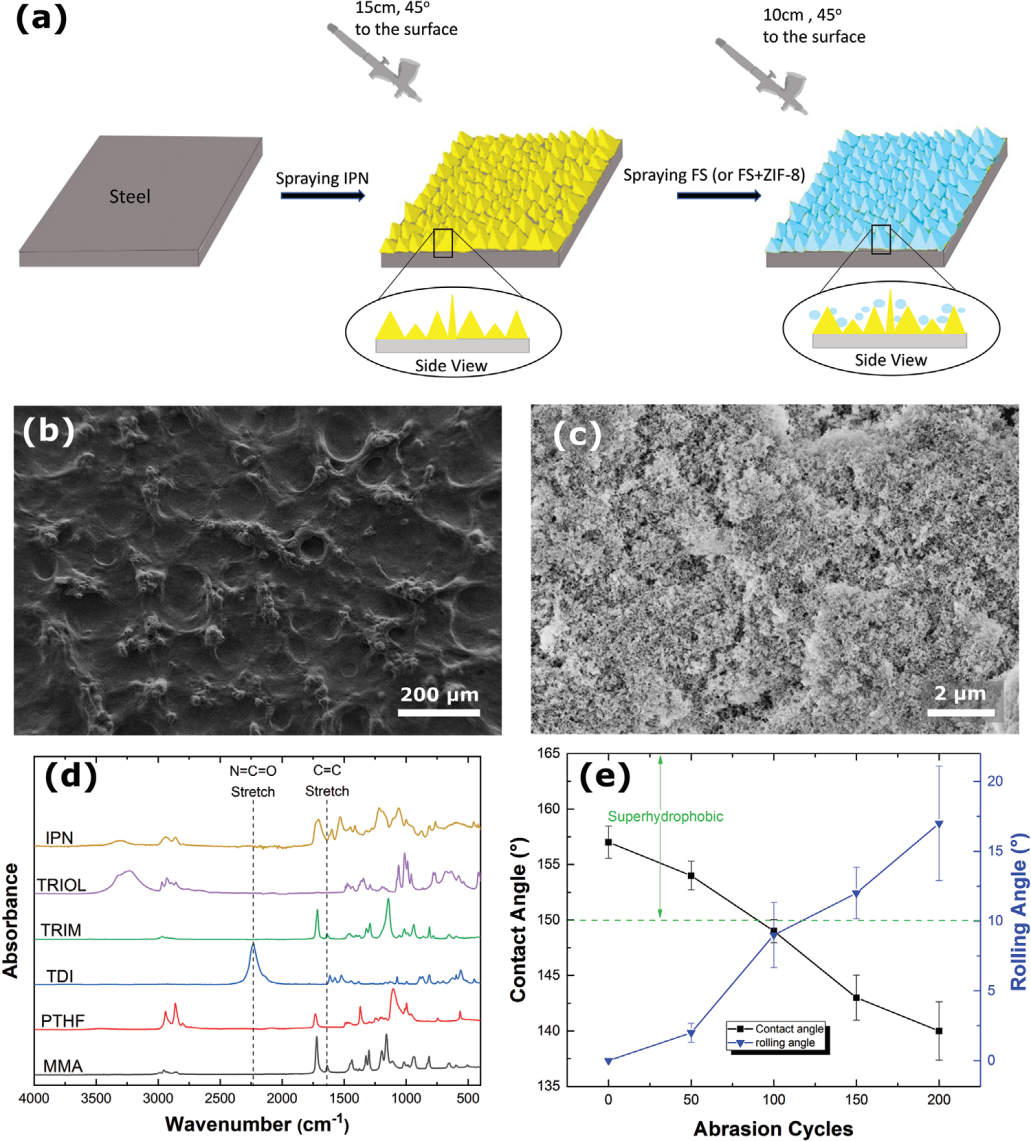
Characterization of the water‐repellent coating. a) Illustration showing the fabrication of the coating. b) SEM images of spray‐coated IPN showing the microtextured morphology. c) SEM images of the IPN‐FS spray‐coated coating show the nanorough morphology of the water repellent coating. d) FTIR spectroscopy of IPN and its raw constituent chemicals. e) The plot shows the static water contact angle and rolling angle versus the number of abrasion cycles (*n* = 27).

**Figure** **2**a illustrates the fabrication of the water repellent coating. Scanning electron microscope (SEM) images of IPN and IPN + FS are shown in Figure 2b,c. IPN provided a micrometer‐scale surface structure (Figure 2b) to which FS nanoparticles are integrated (Figure 2c) resulting in a superhydrophobic water repellent coating. The spray deposition parameters including spraying distance, spraying angle, and the IPN‐curing time (time between spraying IPN and FS) were optimized to improve the integration and the abrasion resistance of the coating. SEM images of IPN and FS at higher magnifications can be found in the Supporting Information (Figures S3 and S4, Supporting Information). The smoothness of the coating was estimated using White Light Interferometry (Figure S5, Supporting Information). The IPN + FS layer (the final water repellent coating) recorded a roughness value of 165.93 ± 63 nm and an average thickness of 2.67 ± 0.42 *μ*m. Spectroscopic analysis of the suspension of IPN is shown in Figure 2d. The loss of the 2235 cm^−1^ N═C═O isocyanate stretch band, 3227 and 3492 cm^−1^ —OH stretch bands belonging to PTHF and TRIOL, respectively, and the formation of 3300 cm^−1^ —NH stretch band confirms the completion of PU reaction.^[^ ^16^ ^]^ The loss of 1637 cm^−1^ C═C stretch band confirms the completion of the PMMA reaction.^[^ ^16^ ^]^ Figure 2e and Table S2 (Supporting Information) show the static water contact angle of the coating measured to be 157 (± 0.47°) and the rolling angle to be 0°. Figure S6 and Movie S1 of the Supporting Information show the lotus leaf effect of our coating applied on a textured surface. This along with the conformality of our contact angle values demonstrates the uniformity of the coating over the surface. Figure 2e shows the effect of abrasion on the static and rolling water contact angles of the coating and Figure S7 of the Supporting Information shows the instrument we used to test the abrasion resistance. Statistically, the water repellent coating was able to maintain superhydrophobic contact angles up to 100 cycles of rotary abrasion under a load of 250 g. After 100 cycles, there is a reduction in static contact angles. As the abrasion cycle increases, the FS nanostructures are slowly broken away and after a certain number of cycles (here 100), abrasion starts ripping away the embedded nanoparticles from the IPN surface, leaving a heterogeneous surface of IPN and FS. Some of the embedded FS stays until 200 cycles of abrasion, leading to the hydrophobic static contact angles shown in Figure 2e. The robustness of the coating is owing to the presence of IPN (composed of 64.75% of PU, a sticky polymer, see the Experimental Section) that sticks to the substrates and also holds the nanoparticles sprayed subsequently. The curing time of IPN and the spray parameters were optimized to get the best performance under abrasion. This durability effectively prevents the interaction between pathogens present in liquids and the underlying coating surface by rolling off landing droplets or avoiding their adhesion and transfer from hydrated or humid host environments such as the human skin.

### 2.2 Bacterial Pathogen Shielding Performance and Mechanism

**Figure 3:**
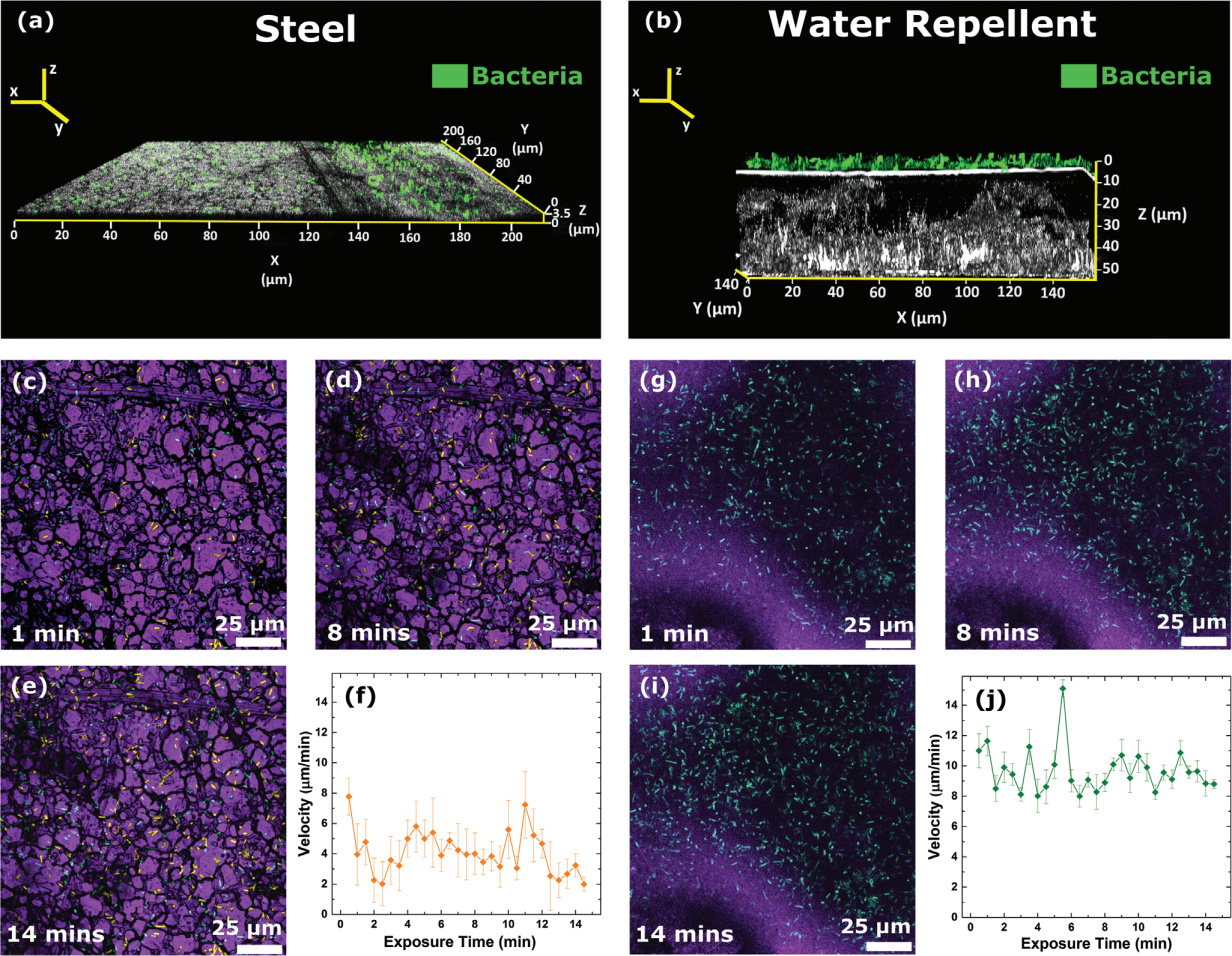
Surface shielding mechanism. a,b) CLSM orthogonal projection of bacteria on steel and water repellent surfaces, respectively. On the water repellent coating, the plastron layer prevents bacteria from reaching and adhering to the surface. c–e) Snapshots from Movie S2 of the Supporting Information show the behavior of bacteria on the steel surface. Adhering bacteria are false colored to yellow and green represents moving bacteria at that particular time‐point. g–i) Snapshots from Movie S3 of the Supporting Information showing the behavior of bacteria on the water repellent coating. The substrate surface is colored purple for contrast. f) Plot showing the velocity profile of bacteria on the bare steel surface, *n* = 15 and j) plot showing the velocity profile of bacteria on water repellent surfaces, *n* = 15.

We used bacteria to evaluate the shielding performance against larger and heavier pathogens than a virus and to gain direct insights into the shielding kinetics of our water repellent coating. Figure S8a of the Supporting Information shows an illustration of the initial interface between bacteria suspended in a liquid and the coating surface, and its time evolution. A continuous air film, denoted as the plastron layer, is initially formed at the solid–liquid interface preventing contact between bacteria and the coating surface. Over time, the plastron layer is removed via the formation and release of air bubbles, induced by the liquid pressure above the coating. These newly immersed coating surfaces become available for bacteria adhesion and eventually result in the formation of a biofilm. To investigate the specific shielding kinetics of our multiscale rough coating, a droplet of bacterial suspension, in its exponential growth phase, was placed and kept on the coating surface and other noncoated materials (bare steel) were used as a comparison (see the Experimental Section; Figure S9a, Supporting Information). **Figure** **3**a,b shows in‐operando in situ confocal laser scanning microscope (CLSM) images of a green fluorescent, bacteria droplet suspension on a bare steel plate and a steel plate coated with the water repellent coating respectively. On the bare steel surface, the bacteria immediately settle and start the surface adhesion process (Figure 3a). In stark contrast, on our coating, the bacteria suspension keeps floating above the plastron layer (white horizontal line shows the liquid meniscus), without being able to reach the underlying surface (Figure 3b).^[^ ^24^ ^]^ Figure 3b also demonstrates the characteristic lotus leaf properties of the coating, where the plastron or the air layer occupies the gaps between the rough protrusions. On the steel surface, the liquid immediately wets the surface and spreads out, and shows no clear liquid meniscus. A slightly tilted orientation of Figure 3b is shown in Figure S10 of the Supporting Information.

To gain further insights into the surface colonization mechanism, a droplet of bacterial suspension was kept on a bare and coated steel surface and the cell behavior was tracked with CLSM (the Experimental Section; Movies S2 and S3 and Figure S9a, Supporting Information). Bacteria possess hair‐like structures on their cell wall, denoted as flagella, which aid them in exploring microscale topography and facilitate adhesion to a surface. Between a solid surface and liquid medium, bacteria exhibit two types of motility, namely, swarming and swimming.^[^ ^25^ ^]^ Swarming is a behavior mediated by flagella and is found on solid surfaces. Here bacteria attach to a solid surface with the help of their flagella and display a characteristic rotational motion. On the bare steel surface, the bacteria displayed such nanoscopic vibrations around an equilibrium position (Movie S2, Supporting Information).^[^ ^24^ ^]^ Swarming and biofilm formation are antagonists to each other as the former is a dynamic surface‐bound motion and the latter represents sessile colonies of bacteria. This means that bacteria will continue to swarm on a surface until they find a suitable location to adhere to. After this, biofilm formation ensues. Swimming is the flagella assisted motion of bacteria in a liquid characterized by the collective commotion of planktonic cells, as seen on the coated surface (Movie S3, Supporting Information). Hence the presence of our coating prevents the surface‐bound motility behavior of bacteria since the plastron layer masks the solid surface underneath. Bacteria float above the plastron layer and are unable to detect favorable sites to attach to.

Figure 3c–e,g–i shows a time sequence of 15 min of CLSM tracking (Figure S9a, Movies S2 and S3, Supporting Information) highlighting the dynamic response of the bacteria in the droplet suspensions on the bare steel and the water repellent coating. Adhered bacteria were identified by lack of displacement and are false colored in yellow as shown in Figure 3c–e. The enlarged images of panels in Figure 3c–e can be found in Figures S11–S13 of the Supporting Information. The average motion of the bacteria on both surfaces was quantified by their average displacement between each time point (Figure 3f,j). Notably, bacteria cells on the bare steel surface had a significantly (*p* < 0.001) lower average velocity of 4.09 (± 1.42) µm min^−1^ than those on the water repellent surface (9.66 (± 1.43) µm min^−1^).

Biofilm formation on an abiotic surface, such as the bare steel here, starts with a reversible primary adhesion stage followed by an irreversible secondary adhesion stage. The primary stage is greatly influenced by hydrophobic interactions between the cell and the surface.^[^ ^26^ ^]^ Our in situ analysis (Movie S2, Supporting Information) indicates that several bacterial cells on the bare steel surface have already entered the secondary stage, showing no translational movement, and firmly adhering to the surface. By contrast, on the water repellent surface (Movie S3, Supporting Information), no bacteria cells lacking motion could be identified, indicating prevention of the primary adhesion stage.

### 2.3 Qualitative and Quantitative Evaluation of the Shielding Performance

**Figure 4:**
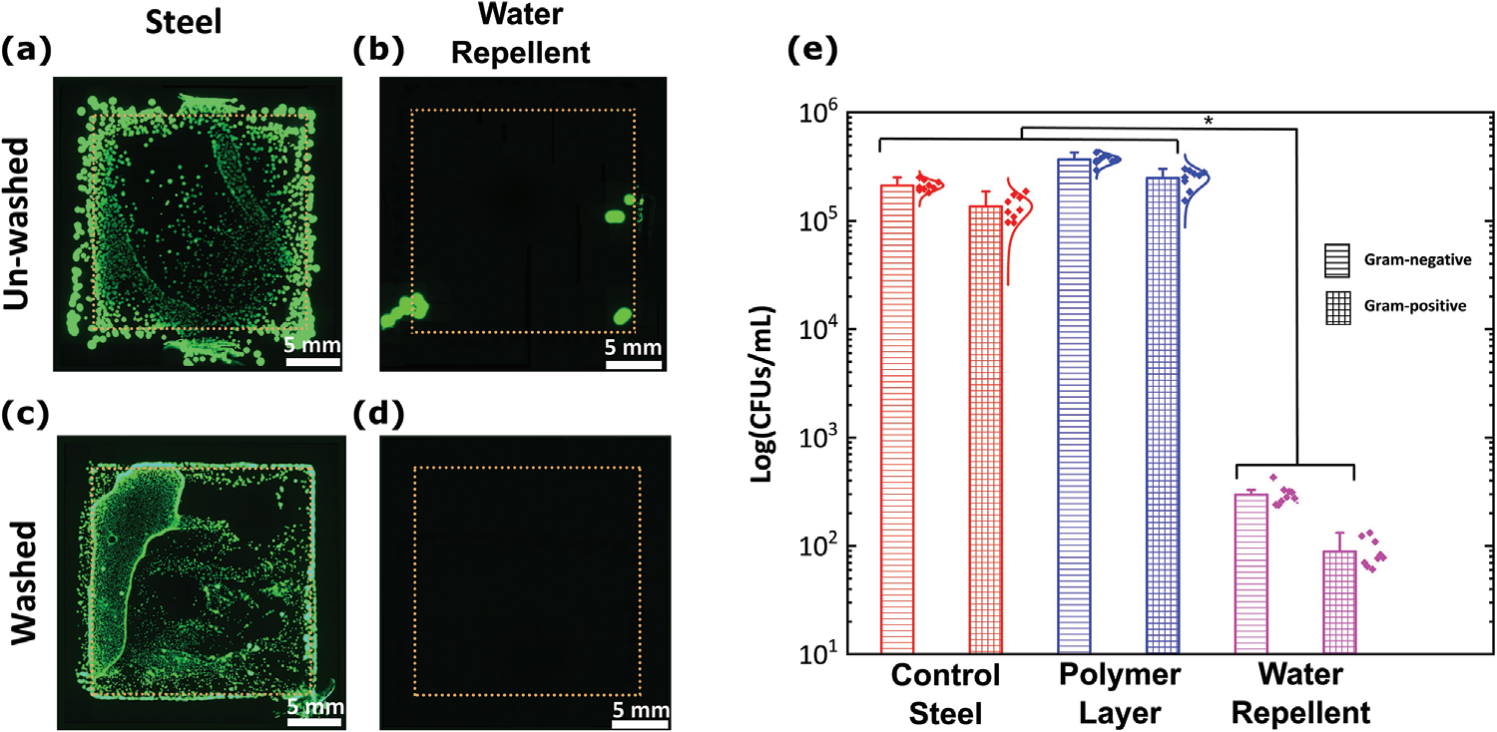
Surface shielding performance against bacteria. a–d) Axio‐observer images of the agar imprints of control‐steel and water repellent surfaces showing qualitatively the relative extent of adhesion under unwashed and washing conditions. e) Plot showing the serial dilution data. The water repellent surfaces resisted 99.99% of bacteria compared to the steel surface. (* represents *p* < 0.01 in a one‐way ANOVA test, *n* = 9.) Imaging experiments were repeated three times.

To attain a quantitative and qualitative assessment of the effectiveness of our surface structure in preventing bacterial adhesion, bare and coated steel substrates were immersed in bacterial culture and stamped on agar plates (see the Experimental Section; Figure S14, Supporting Information), with or without a prior washing step in phosphate buffered saline (PBS). **Figure** **4**a,c shows Axio‐observer microscopy of the agar plates stamped with the washed and the unwashed bare steel substrates. Notably, both samples resulted in the formation of a square‐shaped layer of green fluorescing bacterial colonies, too densely packed to be counted. In stark contrast, the agar plates stamped with the unwashed water repellent coatings show only 12 (± 5) colonies (Figure 4b). Stamping the water repellent coatings after washing resulted in very few bacterial colonies, in the range of 2 (± 2) (Figure 4d). We attribute the small number of bacterial colonies, transferred from the unwashed coatings to the agar plate, to loosely adhered bacteria micro/nanodroplets. Such loosely adhered bacteria may originate from micrometer‐scale defects within the coating or from bacteria adhesion to dust and other contaminants particles that may have been floating over the plastron layer. While stamping, these particles are transferred to the agar plate along with their bacteria load resulting in the formation of a few colonies. This is confirmed by the significant decrease in bacteria colonies upon a gentle washing step (Figure 4d), indicating the absence of biofilm formation. Figure S15 of the Supporting Information shows photographs of agar plates stamped with specimens exposed to both Gram‐negative and Gram‐positive bacteria.

To further quantify the efficacy in preventing bacterial adhesion, serial dilution of the residual PBS used to wash the samples after exposure to bacteria in their log growth phase was undertaken (Figure S14, Supporting Information). Figure 4e shows that the bare steel samples resulted in 2.1 × 10^5^ (± 2.1 × 10^4^) and 1.4 × 10^5^ (± 3.1 × 10^4^) CFUs mL^−1^ colonies for Gram‐negative and Gram‐positive bacteria strains, respectively. Our coating successfully prevented up to 99.85% and 99.94% of the Gram‐negative and Gram‐positive bacteria strain growth (significantly different with a *p*‐value of less than 0.01 in a one‐way ANOVA test) with only 297 (± 56) and 89 (± 24) CFUs mL^−1^, respectively. To validate the essential role of the water repellent texture, control samples consisting of the same polymer coating without the FS layer were also investigated. The latter resulted in slightly higher CFUs mL^−1^ values than the bare steel surface, confirming that the polymer layer does not contribute to a decrease in bacterial adhesion (Figure 4e).

### 2.4 Role of Microscale Coating Defects on Pathogen Adhesion

**Figure 5:**
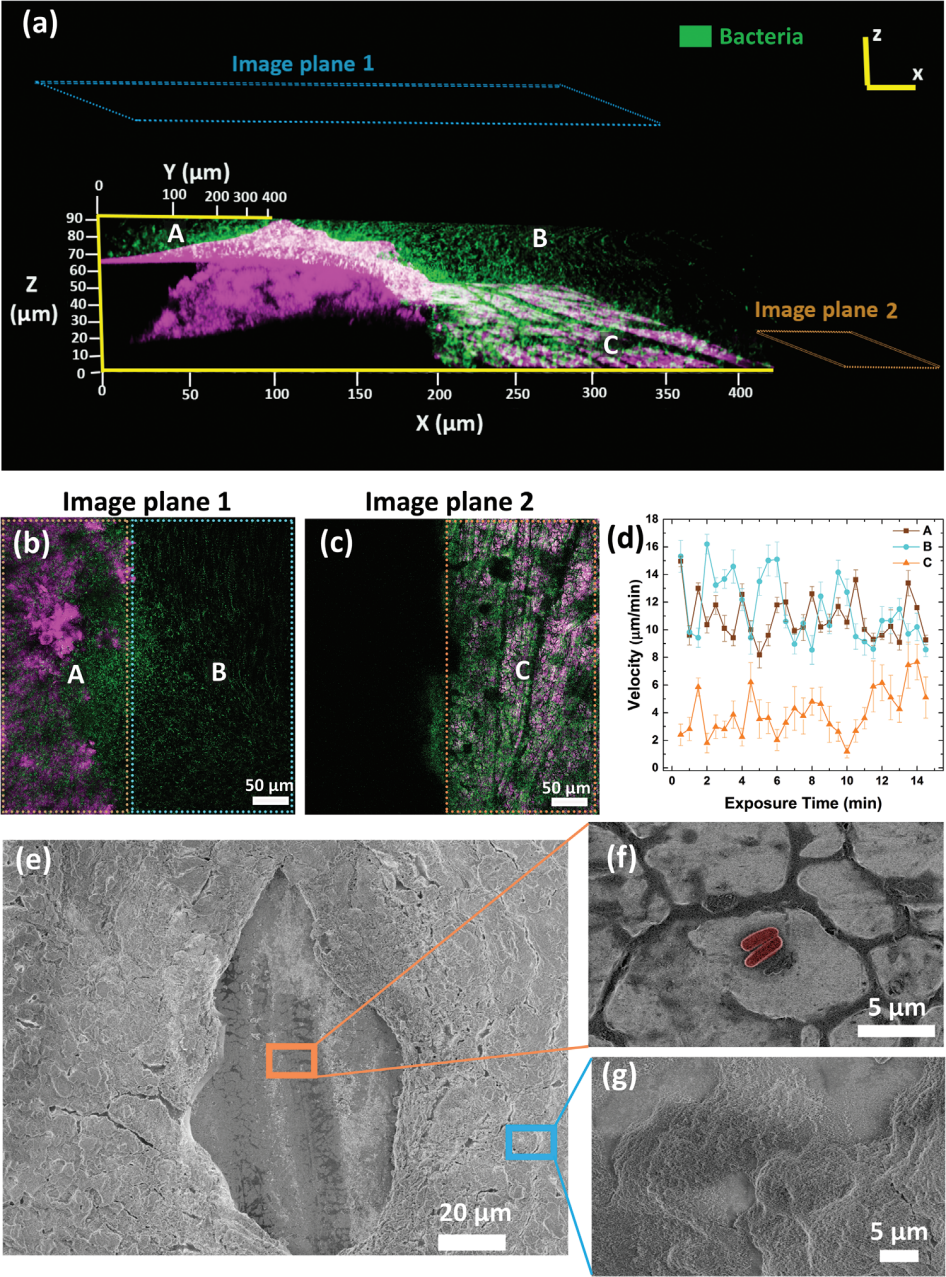
Bacterial adhesion on point defects. a) Orthogonal projection of the coating‐defect interface with bacteria on it. The two image planes are marked. The behavior of cells overtime on these planes is shown in Movies S4 and S5 of the Supporting Information. b,c) Screenshots of the coating‐defect interface captured from Movies S4 and S5 of the Supporting Information. A, B, and C represent three regions where the cell‐surface interactions are studied. d) Velocity profiles of bacteria on regions A, B, and C. e–g) SEM images of a defective coating after exposure to bacterial culture for 10 min. f) The defect is colonized by bacteria, whereas (g) the nondefective area has no bacteria attached to it.

To determine the role of microscale defect sites on the coating on pathogen adhesion, the water repellent coating (FS + IPN) was removed in a quasi‐elliptic area of 62.8 µm^2^, exposing the underlying steel surface. A droplet of bacterial suspension was kept over the defective region of the coating and its intact water repellent surrounding (Figure S9a, Supporting Information). The interface between the microscale defect and the water repellent region was continuously in situ imaged by CLSM. **Figure** **5**a shows an orthogonal projection of the same defect‐coating interface on which the bacterial droplet is anchored. Over the intact water repellent surface (region A, image focal plane 1), a plastron layer with an above floating cloud of bacteria is observed and bacteria display swimming motion, as seen from Movie S4 of the Supporting Information. The plastron layer disappears on the defective area. The latter area consists of two parts (B, C) with corresponding image focal planes 1 and 2, respectively, that are imaged, as shown in Figure 5b,c. Region B corresponds to the bulk of the droplet right above the defective region where swimming motion is observed (Movie S4, Supporting Information; Figure 5b). Region C corresponds to the surface of the defect, which is essentially the surface of steel as the coating is removed from that area. Using image plane 2, we tracked the motion of bacteria and found it to be showing the characteristic surface‐bound motion of attaching to the surface and rotating using their flagella (Movie S5, Supporting Information). The velocity profiles of the cells in regions A, B, and C are shown in Figure 5d. This is clearly in agreement with the distribution we obtained in Figure 3f,j. Regions A and B register much higher values and lower errors compared to C (Figure 5d). Figure 5e shows an SEM image of the defective area of the coating, where attached bacteria can be seen when zoomed in Figure 5f. No bacteria could be found on the pristine part of the coating (Figure 5g). We also performed CLMS imaging of our water repellent surface before after subjecting them to abrasion with the methodology depicted in Figure S9b of the Supporting Information. The results are shown in Figure S16 of the Supporting Information. The average thickness of the plastron reduces as the number of abrasion cycles increases. After 200 cycles of abrasion, the plastron thickness becomes negligible, substantiating the abrasion results we showed in Figure 2e.

### 2.5 Failure Kinetic of the Water Repellent Shielding Mechanism Underwater

In addition to the presence of defects on the coating, the long‐term stability of the plastron layer underwater is a key factor to prevent bacteria and other pathogens adhesion to the surface. Previous studies report that the plastron layer on superhydrophobic surfaces is lost within 1–1.5 h of continuous immersion in water (Table S1, Supporting Information). Here, to investigate the stability of the plastron layer on our surface structure, coated steel substrates were immersed in liquid bacterial culture at a depth of 2.5 cm, and the water–surface interface was continuously imaged by CLSM throughout 8 h (see the Experimental Section; Figure S9b, Supporting Information). Figure S8 of the Supporting Information illustrates the disintegration of plastron over time underwater and how it leads to bacterial colonization.

**Figure 6:**
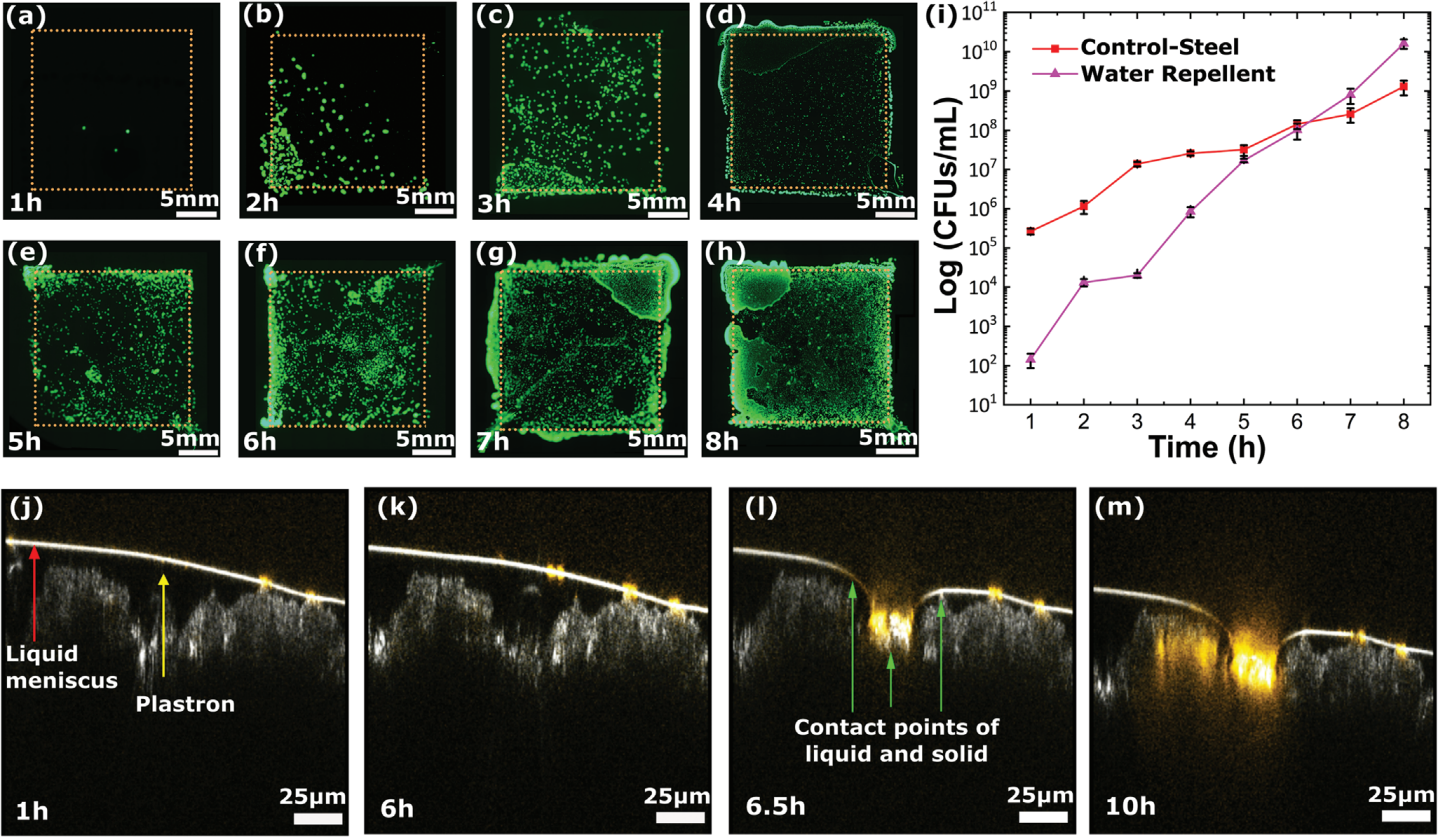
Failure kinetic of water‐repellent shielding mechanism and performance of the secondary shielding mechanism. a–h) Axio‐observer images of the agar plate imprints corresponding to water repellent surfaces submerged in bacteria solutions for 1 h to 8 h. Up to <6 h immersion, a decrease in colonization concerning the bare steel coating is observed. i) Serial dilution data of the long immersion experiment, *n* = 9. j–m) Snapshots from Movie S5 of the Supporting Information show the plastron layer disruption over time.

**Figure** **6**a–h shows images with green fluorescent bacteria on agar plates stamped with the water repellent steel substrates as a function of their immersion time from 1 to 8 h. Notably, for up to 4 h of continuous immersion very few bacterial colonies can adhere to the substrate (Figure 6a,b). For exposures of 4 to 6 h, a rapidly increasing number of bacteria colonies were observed (Figure 6c–e). While longer time results in complete colonization of the bacteria‐challenged area (Figure 6f–h). The immersion depth is an important parameter often overlooked in plastron‐stability studies (Table S1, Supporting Information).^[^ ^27^ ^]^ Following extended submersion of the coating, the liquid pressure on the plastron layer causes its gradual dissolution. As the plastron continues to degrade and thin, it exposes parts of the underlying coated surface to the bacteria allowing for their adhesion. We have observed that the multiscale roughness of our coating structure increases the stability of the plastron layer up to 4 h. This is significantly longer than previous studies on water repellent coatings reporting plastron loss within 1 to 1.5 h (Table S1, Supporting Information).

For exposures of 4 to 6 h, a rapidly increasing number of bacteria colonies were observed (Figure 6c–e). While longer time results in complete colonization of the bacteria‐challenged area (Figure 6f–h). This trend was quantified by serial dilution data showing that up to 4 h a significant reduction of the number of bacteria colonies is observed compared to the bare steel surface (Figure 6i; Table S3, Supporting Information). Notably, the water repellent coatings were able to decrease the number of bacteria colonies by 99.8% for up to 4 h continuous immersion. This indicates a plastron layer stability in the range of 3–4 h. Upon 5–6 h continuous immersion, the bare and coated steel samples resulted in similar bacteria colony densities (Table S3, Supporting Information). Notably, for immersion times of 7 h and longer, the water repellent coatings displayed an increasingly larger number of bacteria colonies (Table S3, Supporting Information). This is attributed to the micro and mesoscale roughness of the water repellent coatings that provide a larger area for bacteria adhesion upon dissolution of the plastron layer. Previous studies on the use of water repellent coatings to prevent bacteria adhesion have not been able to achieve comparable reductions in bacteria colonies for such an extended period of immersion time (Table S1, Supporting Information). Significant reduction in bacteria colonies was reported only for significantly shorter immersion times of 1 h or less. One of the most recent studies that analyzed the plastron collapse of water repellent coatings observed less than 90% reduction for 1 h.^[^ ^27^ ^]^

To gain insights into the mechanism determining the longer stability of the plastron layer on our water repellent surfaces, in situ studies were conducted by immersing our coating in a column of deionized water colored with rhodamine B and imaging using CLSM (Figure 6j–m; Figure S9b and Movie S6, Supporting Information). The collapse of the plastron layer was captured by the formation of a series of sequential contact points with the surface structure (Figure 6k,l). This process of plastron collapse rapidly accelerates after 6 h when the release of air bubbles trapped in the occlusions of the surface structures is observed (Movie S6, Supporting Information). Subsequently, the surface becomes completely wet as highlighted by the dye accumulation and the correlated increase in the fluorescence intensity of the dye (Figure 6l,m). The lifetime of the plastron layer is also a function of the surface roughness, and thus in the case of hierarchically rough surfaces, the plastron layer lifetime depends on the local topography of the coating.^[^ ^28^ ^]^ The multiscale surface roughness of our coatings results in a thicker plastron layer. Consequently, it takes a longer time for its complete collapse and disappearance (see Note S1 of the Supporting Information for more characterization and discussion).

### 2.6 Secondary Surface Shielding Mechanism: Dual Functional Coating

Our water repellent coating protects against bacteria (and viruses as well, demonstrated in the next section) until the failure of the plastron layer. However, despite providing longer stability than previous reports on water‐repellent surfaces (>4 h of continuous submersion), we acknowledged that the eventual dissolution of the plastron layer limits the breadth of applications, for instance, in marine environments, membrane systems, or bathrooms, where an anti‐biofouling surface that could tolerate extended submersion is required. To this end, we have exploited the antimicrobial and hydrophobic properties of ZIF‐8 nanograins to impart a secondary shielding mechanism to the water repellent coatings, the newly formed coating now called a dual‐functional coating.

### 2.7 Material Characterization of the Dual‐Functional Coating

**Figure 7:**
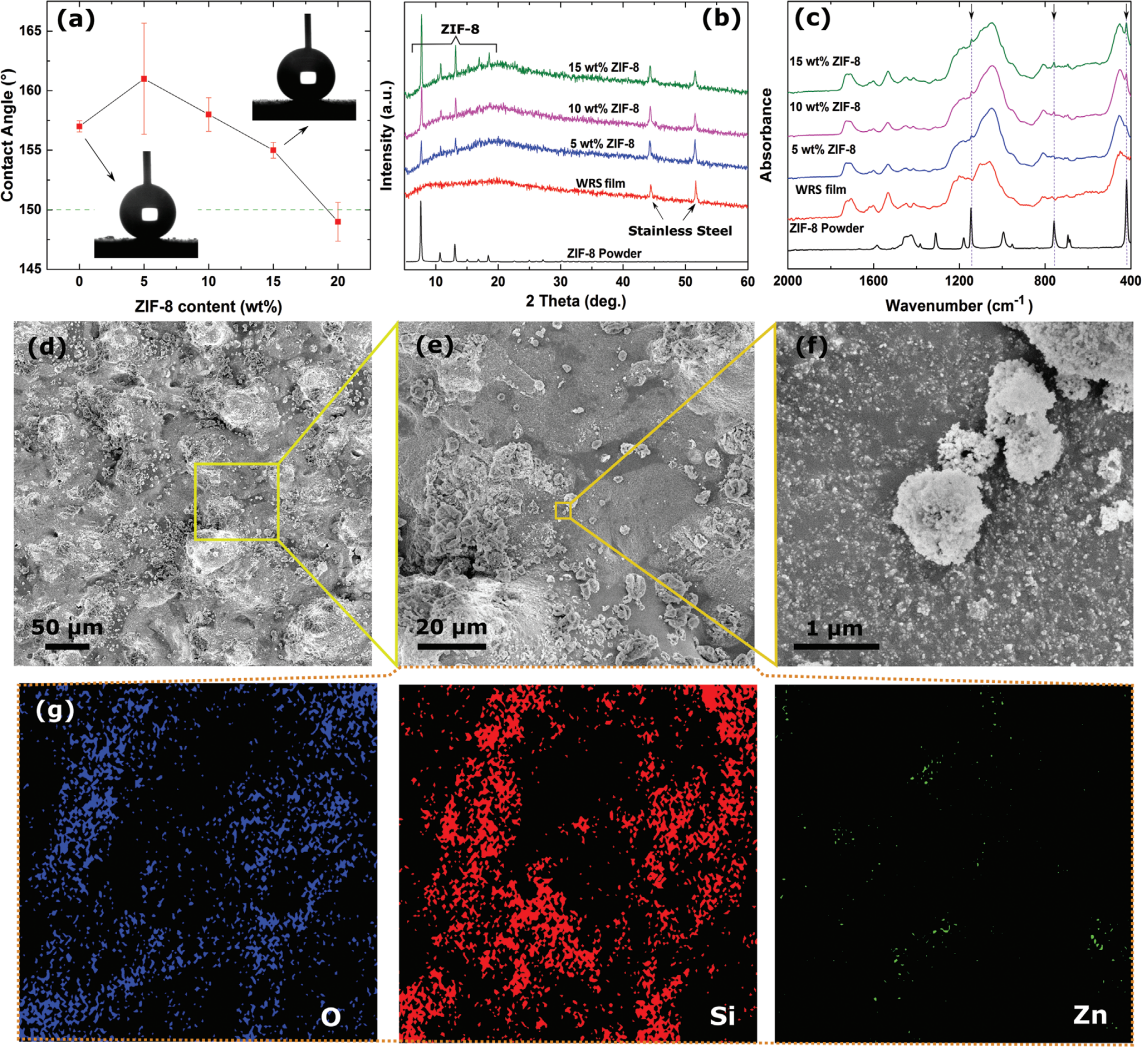
Material characterizations of the dual‐functional coating. a) Contact angles of water repellent coating and the samples containing ZIF‐8 powders (5–20 wt%). b) XRD patterns, and c) FTIR spectra of the coatings (water repellent coating and the samples containing 5–15 wt% of ZIF‐8 powders) on stainless steel in comparison with ZIF‐8 powders. d–f) SEM images of the coating containing 15 wt% of ZIF‐8 powders at different magnifications, and g) EDX elemental mapping of the coating showing the distribution of oxygen, silicon, and zinc elements.

Figure S17 of the Supporting Information shows an SEM image of ZIF‐8 powders. The absence of hydrophilic functional groups in the HmIm makes ZIF‐8 inherently hydrophobic, contributing to the coating superhydrophobicity.^[^ ^29^ ^]^ The spontaneous release of Zn^2+^ ions from ZIF‐8, due to its gradual decomposition in a broad range of pH values bears promise for its use as an antimicrobial agent.^[^ ^22^, ^30^ ^]^ Here, the presence of ZIF‐8 is aimed at killing bacteria that may adhere to the coating due to damage to its structural integrity and thus localized loss of superhydrophobicity, evaporation of their liquid shell environment before removal from the surface, or loss of the air layer due to prolonged immersion in a contaminated liquid (Figure 1b). We added ZIF‐8 into our FS solution at 5, 10, 15, and 20 wt % and sprayed over the IPN layer following the spray parameters as in Figure 2a. 5 wt% ZIF‐8 coating registered the highest contact angle value of 161°, 10 wt% with 158°, 15 wt% with 155° and 20 wt% with 149° (Table S2, Supporting Information; **Figure** **7**a). The rolling angles were 0° for all samples except 20 wt%, where it went above 10°. We hypothesize that the presence of ZIF‐8 in the coating until a certain amount creates an additional degree of roughness. However, once the limit is crossed, the hydrophobic ZIF‐8 particles start being exposed on the surface, resulting in a reduction of the contact angle. For 20 wt% ZIF‐8, even though the angle was close to the characteristic superhydrophobic boundary, the increase in rolling angle was pinning the droplet on the surface. Hence, we decided to eliminate 20 wt% for the other characterizations. Since we used the same IPN in the coating that holds the nanoparticles sprayed later, the durability of the dual‐functional coatings remained similar to the water repellent coating. In XRD analysis, all the ZIF‐8 coatings record strong peaks at 2*θ* = 7.30°, 10.35°, 12.70°, 14.80°, 16.40°, and 18.00° that indicates the presence of lattice planes (100), (200), (211), (310), and (222), respectively (Figure 7b).^[^ ^31^ ^]^ In Fourier transform infrared (FTIR) analysis, the strong organic character at 421 cm^−1^ observed shows the presence of zinc‐nitrogen stretching that occurs between zinc and nitrogen of the HmIm organic linker (Figure 7c).^[^ ^32^ ^]^ Microstructural and elemental analysis of the coating containing 15 wt% of ZIF‐8 powders is presented in Figure 7d–f. Considering the average particle size of the ZIF‐8 powders (200–400 nm), it seems that the MOF particles are covered by FS as shown in Figure 7d–f. EDX elemental mapping of the coating (Figure 7g) confirms the presence and dispersion of zinc element (from the ZIF‐8 structure) in this dual‐functional coating.

### 2.8 Antibacterial Performance of the Dual‐Functional Coating

**Figure 8:**
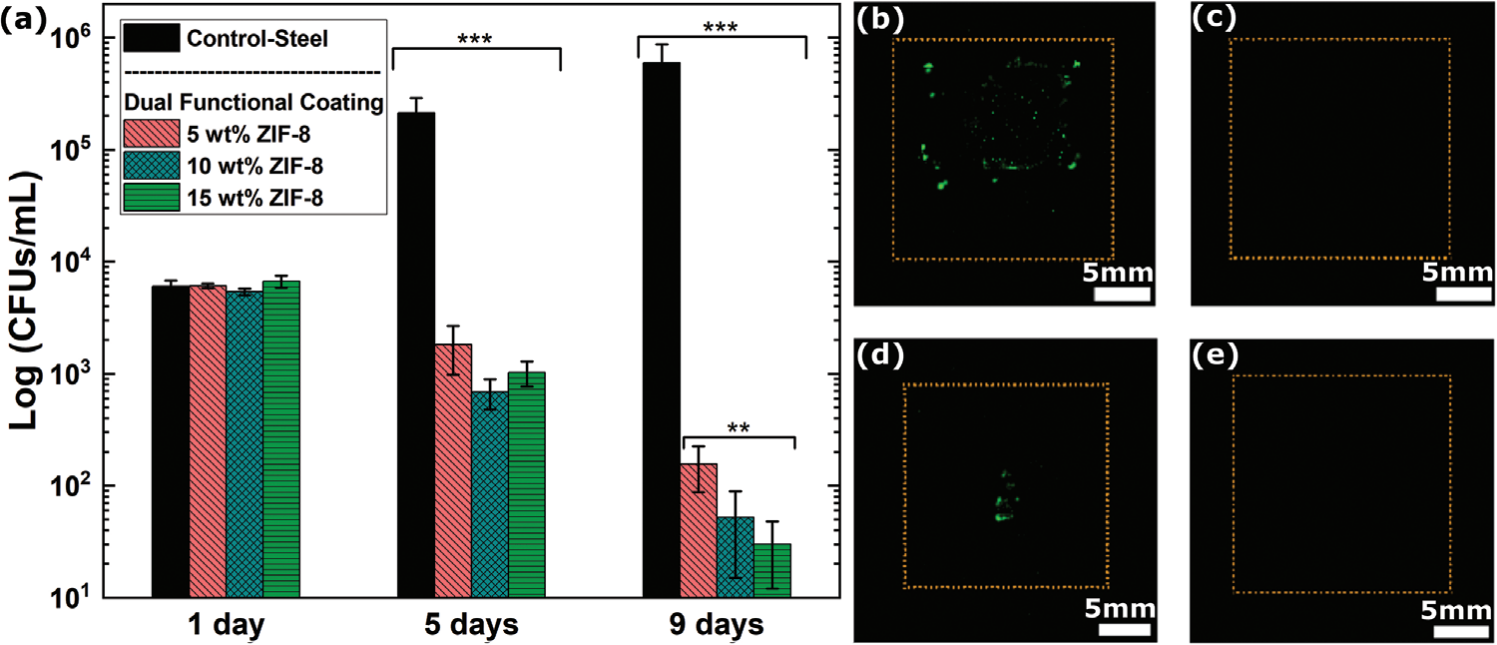
Antibacterial characterization of the dual‐functional coating. a) Plot showing the serial dilution data of the dual‐functional coatings containing ZIF‐8 nanograins after 1, 5, and 9 days continuous immersion in a highly concentrated bacteria solution (** represents *p* < 0.01 and *** represents *p* < 0.001 in a one‐way ANOVA test, *n* = 6). Results of the scratch‐experiment showing Axio‐observer images of the agar imprints of b) control‐steel, c) pristine water repellent coating, d) scratched water repellent coating, and e) scratched dual‐functional coating containing 15 wt% of ZIF‐8. All the imaging experiments were repeated three times.

As the dual‐functional coatings have superhydrophobic properties similar to the water‐repellent coating, the antibacterial characterizations, performed in Figures 3 and 4, stand valid for the former as well. The capability of this surface composition to eliminate bacteria proliferation even in the event of dissolution of the plastron layer was demonstrated by long‐term immersion of coated and bare steel substrates in a 5 cm column of a solution containing 10^4^–10^5^ bacteria cells mL^−1^ (**Figure** **8**a). After 1 day of immersion, there was no statistical difference between the noncoated and coated substrates. However, after 5 days of immersion, all the ZIF‐8 containing coatings revealed a significant reduction in the cell counts of 2–3 orders of magnitude (Figure 8a). More specifically, while on the bare substrates the bacteria colonies increased from 6 × 10^3^ (± 7.5 × 10^2^) to 2.1 × 10^5^ (± 7.5 × 10^4^) with immersion time increasing from 1 to 5 days, on the coated surfaces the bacteria colonies decreased from 6.1 × 10^3^ (± 2.6 × 10^2^), 5.4 × 10^3^ (± 3.7 × 10^2^), 6.7 × 10^3^ (± 8.2 × 10^2^) to 1.8 × 10^3^ (± 8.4 × 10^2^), 6.9 × 10^2^ (± 2.1 × 10^2^), 1.1 × 10^3^ (± 2.6 × 10^2^) in the same time interval for the 5, 10, and 15 wt% ZIF‐8 content, respectively. The antimicrobial effect of the ZIF‐8 became more accentuated with increasing immersion time with bacteria colonies further decreasing on the coatings after 9 days of continuous immersion (Figure 8a). At such prolonged interaction between bacteria and coated surfaces, the impact of the ZIF‐8 content became measurable, with the 15 wt% ZIF‐8 containing coatings showing the smallest number of colonies of 3 × 10^1^ (± 1.8 × 10^1^) versus the 1.6 × 10^2^ (± 6.9 × 10^1^) of the 5 wt% ZIF‐8 containing coatings and the 5.9 × 10^5^ (± 2.8 × 10^5^) of the bare surfaces (Figure 8a). Due to these observations, 15 wt% ZIF‐8 containing coating is used for further characterizations and will be called dual‐functional coating wherever not explicitly stated.

We further evaluated the efficacy of this antimicrobial mechanism to prevent bacteria adhesion in case of physical damage to the coating and subsequent loss of its water repellent functionality using a scratch experiment. A scratch was imparted on the water repellent coatings and the dual functional coating. A droplet containing bacteria culture in the log phase was left on the defect (Figure S18, Supporting Information), upon which the samples were washed with PBS and then incubated for 24 h. Subsequently, the samples were stamped on agar plates, then incubated and analyzed by microscopy (Figure 8b–e). The bare steel samples, used as controls, show significant bacteria adhesion (Figure 8b). The nonscratched water repellent coatings had no bacterial adhesion (Figure 8c) in line with the plastron layer‐based bacterial repulsion mechanism. The scratched water repellent coatings made of pure fluorosilica particles resulted in bacteria adhesion in the proximity of the scratched region (Figure 8d). Notably, the scratched dual‐functional coating containing 15 wt% ZIF‐8 nanoparticles resulted in no bacteria adhesion despite the loss of their water repellent functionality (Figure 8e). We believe this is due to the localized release of zinc ions that prevent or kill the bacteria approaching the surface. We also quantified the release of zinc ions from our dual‐functional coating in immersed conditions (Figure S19, Supporting Information). The results agree with our original estimation of plastron stability to be around 6–8 h (Figure 6; Figure S19, Supporting Information). There is a burst release of ions after this time point, suggesting the loss of plastron. The ionic concentration increases and saturates around after 72 h (Figure S19, Supporting Information).

### 2.9 Protecting Surfaces from Viral Contamination

**Figure 9:**
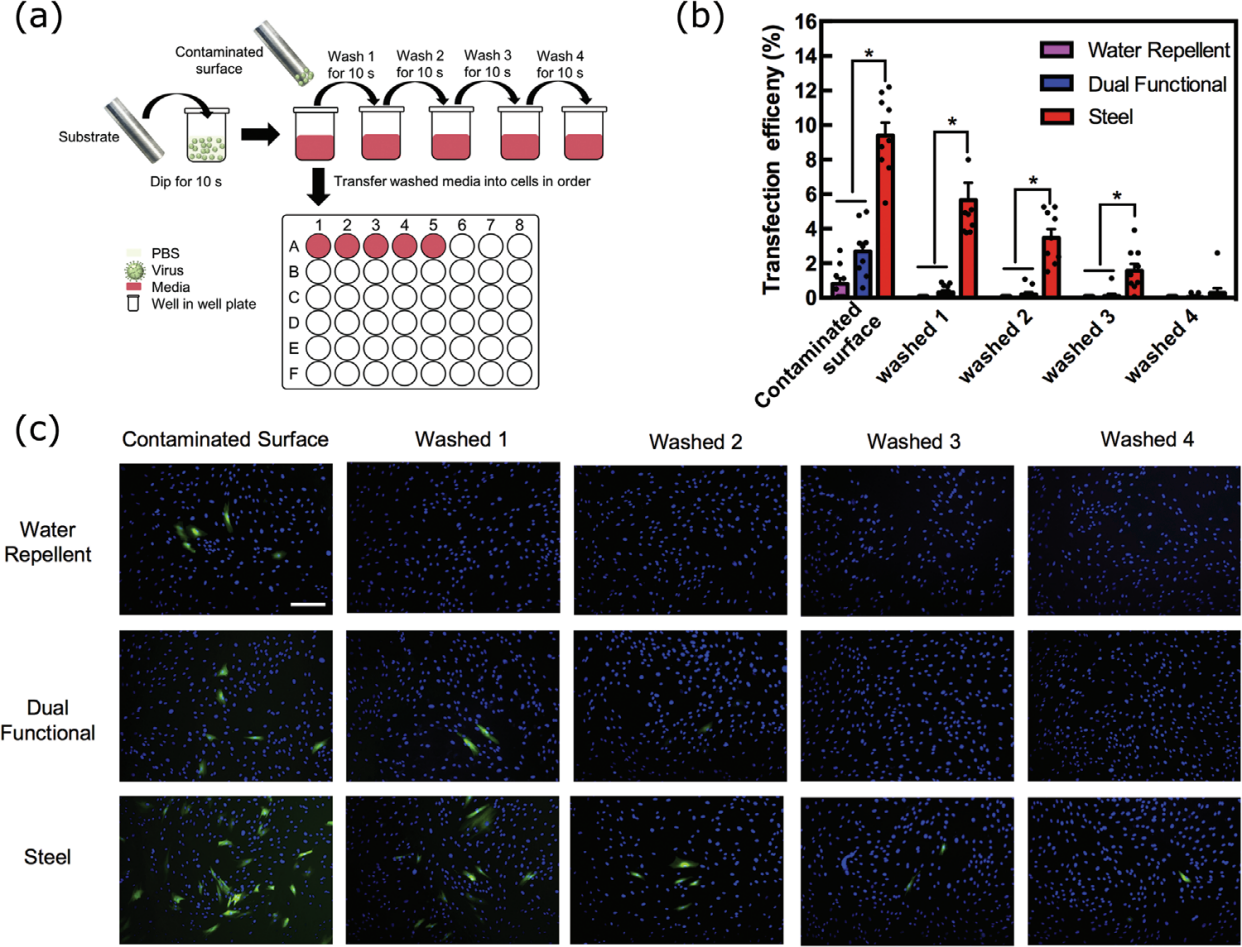
Evaluation of viral pathogen shielding performance. a) Schematic of the experiment, where a metal rod is first dipped in a solution of an EGFP‐expressing lentiviral vector (1 × 107 TU mL^−1^) and then through a series of wash solutions (fresh DMEM). The washes were then tested for virus by incubating with cells and detecting infection by fluorescence microscopy. b) Quantification of numbers of infected cells as counted by microscopy, representing levels of virus contamination. Each replicate is shown as a dot (*n* = 9), the bars and error are mean and SEM, respectively; * represents *p* < 0.01. c) Representative images of cells after incubation with the washes as labeled. Green indicates infection and blue is 4′,6‐diamidino‐2‐phenylindole (DAPI) stained nuclei, scale bar = 100 µm.

We explored the performance of our water repellent and dual functional coating in preventing viral contamination of surfaces. We used a steel rod with significant surface roughness and tested its ability to mediate transmission of viruses in its uncoated state, or coated with our coatings (Figure S20, Supporting Information). For each test, the rod was dipped into a high titre solution of an enhanced green fluorescent protein (EGFP)‐expressing lentiviral vector as a surrogate for infectious viruses. The rod was dipped in a series of inert washing solutions (fresh Dulbecco's modified Eagle's medium (DMEM)) to determine the level of initial contamination and ease of cleaning (**Figure** **9**a; Figure S20, Supporting Information). The first washing solution represents the capacity of the rod to resist viral contamination upon exposure to a high concentration of lentiviral (i.e., human immunodeficiency virus) solution (1 × 10^7^ TU mL^−1^). Analysis of the first washing solution reveals a significant capability of the coatings to prevent viral contamination of the rod (*p* = 0.05, Figure 9b). More than an 11‐fold decrease in cell infection was observed from the bare rod (9.4% (± 0.7%)) to the ones coated with a water repellent surface (0.8% (± 0.3%)). The dual‐functional coating also effectively prevented viral contamination, with a roughly threefold decrease in cell infection compared with bare steel. Remarkably, no virus was detected upon the first wash on the rod coated with the water repellent coating and only a very small amount of virus was detected for the dual functional coating, which was eliminated after the 2nd wash. The reduction in virus between the first two steps (contaminated surface to wash 1) for the dual functional coating was ≈11‐fold, after which detection of the virus became sporadic. By contrast, the bare steel carried virus through all four wash steps and showed a linear decline, with infectivity reducing by around half with each successive wash. The slightly decreased shielding effect in the case of the dual‐functional coating may be due to the exposure of some sub‐micrometer ZIF‐8 domains that are not superhydrophobic.^[^ ^29^, ^33^ ^]^ These domains may provide attachment points for some virions, but these few isolated and loosely adhered particles were easily removed upon a first gentle wash. Importantly, both coated surfaces performed exceptionally well compared with bare steel that was significantly more strongly contaminated and continued to shed infectivity through multiple washes. The virus was quantified in the washing solutions by incubation on a monolayer of susceptible cells followed by fluorescence microscopy to detect and count infected cells (Figure 9c). The water repellent and dual functional coatings had significantly fewer infected cells right after the first wash, compared to the noncoated steel surface (Figure 9c). The observation proves the effectiveness of our coatings in significantly reducing the surface stability of viral particles by promoting their easy removal. As long as the continuous air film is preserved, the coatings due to their nonselective nature will work against both enveloped and nonenveloped viruses.

## 3 Conclusions

Realizing surfaces that are inherently able to resist pathogen contamination is central to healthcare strategies aimed at preventing pandemics. We introduced a dual functional coating capable of shielding the surface from viruses and bacteria. Our dual‐functional coating decreased the adhesion of bacteria by 99.8%, even when continuously immersed for 9 days in highly concentrated bacteria solutions. Importantly, the water repellent mechanism was also functional against the virus, decreasing uptake by 11 times. Complete removal of the virus only required a gentle, single wash, while it was not possible to completely remove the same viruses from an uncoated steel surface even after 4 washes. The ease of fabrication of our coating by a rapid spray process and its compatibility with most materials make it suitable for preventing pathogen contamination of a broad range of high‐touch surfaces in public settings.

## 4 Experimental Section

### Synthesis of IPN

IPN here accommodates a mix of tough characteristics of PMMA and the rubbery nature of PU, as explained in the previous study.^[^ ^16^ ^]^ This layer provides robustness to the overall coating structure by firmly holding the nanoparticles whilst being adhered to the substrate. The polymeric suspension was prepared in a single‐step one‐pot synthesis approach as shown in Figure S1 of the Supporting Information. Acetone (100 mL, Sigma‐Aldrich, HPCL reagent 99.9%) and xylene (100 mL, Univar, 99%) as the solvents were added to a 3‐ neck round bottom flask. Following this, the constituents that form PU. 1,1,1,‐Tris(hydroxymethyl)‐propane (2.2 g, Triol, Sigma‐Aldrich, ≥98%) were added to the solvent mixture and stirred rapidly at 1000 rpm to disperse off the solids. After adding polytetramethylene ether glycol (10 mL, Polyol, Sigma‐Aldrich, M_n_ ≈ 2000) and tolylene‐2,4‐diisocyanate (3.57 mL, TDI, Sigma‐Aldrich, 95%) as the crosslinker, the flask was closed using rubber stoppers and the mixture was left under stirring at 500 rpm for 30 min to initiate the polymerization of PU.

After this, the constituents that form PMMA – methyl methacrylate (10 mL, MMA, Sigma‐Aldrich, 99%), trimethylopropane trimethacrylate (0.472 mL, TRIM, Sigma‐Aldrich, technical grade), and 2,2′‐azobis (2‐methyl propionitrile) (0.304 mL, AIBN, Sigma‐Aldrich, 0.2 m in toluene) as the crosslinker was added to the flask. TDI and Polyol were melted at 50 °C in a drying oven before adding to the flask. To catalyze the reaction, dibutyltin dilaurate (50 µL, DD, Sigma‐Aldrich, 95%) was then added and the flask was sealed and transferred to an oil bath under dark maintained at 60 °C (PMMA polymerizes by a free radical mechanism at this temperature) and stirred for 24 h at 500 pm. Before initiating the reaction, a condenser was attached to one of the necks of the flask to prevent the evaporation of solvents. The suspension obtained postreaction is called the sprayable PU‐PMMA IPN. TDI:TRIOL molar ratio here is 1.5 compared to the previous study where it was 2.41^[^ ^16^ ^]^ and no water was added to the IPN reaction. IPN has a PU:PMMA ratio of 64.75:35.25 and it was storage stable at room temperature showing no inconsistency even after 10 months.

### Synthesis of FS

FS was sprayed after spraying IPN. FS forms the superhydrophobic nanoparticle component that helps in repelling water as well as pathogens. The nanoparticles of FS were prepared by functionalizing fumed silica with organic fluorine precursor as shown in Figure S2 of the Supporting Information. In a round bottom flask, anhydrous chloroform (160 mL, Sigma‐Aldrich, ≥ 99%), and fumed silica powder (4 g, Sigma‐Aldrich, 7 nm) were added and purged with dry nitrogen for 40 min under mild stirring. Trichloro(1H,1H,2H, 2H‐perfluorooctyl)silane (0.95 mL, Sigma‐Aldrich, 97%) was added to the reaction mixture. The flask, necks of which sealed with rubber stoppers was left to progress for 48 h under 500 rpm stirring. After this, obtained FS was washed in 4 cycles of chloroform and then left to dry in a fume hood at room temperature for 48 h. The FS particles formed were then suspended in analytical grade acetone at a concentration of 20 mg mL^−1^ and ultrasonicated for 2 h to form a finely dispersed and sprayable solution.

### Fabrication of the Coating

Stainless Steel 316L (referred to simply as steel in the main text) substrates of 2 × 2 cm^2^ were initially washed in an acetone bath for 15 min and dried inside a fume hood. The dried substrates were then sprayed using artist's acrylic spray guns of 0.3 mm in diameters. 5 mL of IPN was sprayed on each side of the substrate. The substrates were left for curing for 10 min and then sprayed with 5 mL of FS on each side of the substrates to achieve a water repellent coating. For bacterial adhesion assay, the substrates were spray‐coated on their edges as well.

### Material Characterization of the Water‐Repellent Coating

A field‐emission SEM (Zeiss Ultraplus) was used to analyze the surface morphology of the coating. Before the SEM analysis, the samples were sputter‐coated with platinum at 20 mA for 1 min. For SEM analysis with bacterial cells on the surfaces, 2 steps of cell fixation in glutaraldehyde and osmium tetroxide followed by multiple dehydration steps in solutions with increasing ethanol and then critical point drying was performed before imaging.

Chemistry of IPN (polymerization, functionalization, and crosslinking) was analyzed using FTIR‐attenuated total reflectance (Bruker‐Alpha, USA) on all as‐synthesized samples and the prereaction constituents. Scans were performed from 400 to 4000 cm^–1^. Static and rolling water contact angles were measured using a contact angle analyzer (Surface Electro‐Optics, Phoenix – MT (M, A, T). A total of 9 samples were tested. Abrasion analysis of the coating was quantitatively assessed using a rotary platform abrasion tester with two CS‐10 (Calibrase, USA) abrasive wheels resurfaced with 150 grit disks). The disks were loaded 250 g and were rotated at 60 pm. A method is a well‐approved approach with parameters chosen based on the ASTM D4060 Taber standard. The spray parameters mentioned before were optimized to get the best abrasion results. A total of 9 samples with three different regions on each sample were tested. White Light Interferometry (Veeco, Wyko NT9100, USA) with 50–500× magnification and field of view of 1× via the vertical scanning interferometry mode was employed to assess the surface texture of the coating.

### Preparation of Bacterial Cultures

The liquid cultures of *Escherichia coli* (*E. coli*) JM109 (Gram‐negative) and *Staphylococcus aureus* (Gram‐positive) were routinely grown in Luria–Bertani (LB) medium at 37 °C with shaking at 180 pm. For fluorescent microscopy, *E. coli* JM109 cells were transformed with plasmid pCR2.1/T7‐GFP (Addgene) by electroporation to express a GFP and were grown in media containing 100 µg mL^−1^ ampicillin to maintain the plasmid. The overnight cultures were diluted at 1:100 in fresh LB to obtain a log‐phase culture with an optical density at 600 nm (OD_600_) between 0.4 and 0.6.

### CLSM Experiments

To understand the mechanism behind the water‐repellent effect of the coating, the surface interactions of bacteria on a coated and noncoated surface and also the disintegration of this mechanism at different conditions including mechanical wear and underwater exposure, CLSM imaging was employed. Specimens were imaged on a Zeiss Examiner Z.1 microscope fitted with an LSM 780 confocal using ZEN 2012 software (Carl Zeiss, Germany). All images were collected with a W‐Plan‐Apochrom/attic 20 × 1.0 N.A water‐dipping lens. Z series ranging from 70 to 705 µm were all collected at 0.8 µm step intervals. Image format was varied between experiments with 1024 × 1024 (0.22–0.42 µm pixel size) used when the *x*–*y* resolution was deemed more important than speed and reduced to 512 × 512 pixels (0.69–1.19 µm pixel size) when the speed of acquisition was considered to be more important than resolution. Early attempts used a water depth of ≈15 mm, this was subsequently increased to ≈32 mm. Time‐course imaging was varied depending on the experiment and included every 30 min for 23 h, 20 min for 16 h and every 6 min for 15 h for rhodamine experiments through to 6 min intervals over 90 min and 2 min over 150 min for the EGFP *E. coli*. For rhodamine surface wetting studies, a two‐track approach was utilized with surface details obtained in one track using the 561 nm laser, an 80/20 main beam splitter and a detection window of 550–570 nm configured in reflection mode. Rhodamine fluorescence was obtained in the second track using 561 nm excitation, a 458/561 nm main beam splitter and an emission window of 570–695 nm. For GFP tagged *E. coli* studies a two‐channel approach was utilized, excitation was at 488 nm utilizing an 80/20 beam splitter, 488 nm reflection was collected in channel 1 with a detection window of 485–495 nm and GFP fluorescence was collected in channel 2 using a 508–580 nm collection window. Postprocessing, analysis, annotation, and movie creation was performed using a combination of ZEN 2012 software and Fiji/ImageJ2 (Figure S6a, Supporting Information).^[^ ^34^ ^]^ The bacterial cells were tracked for their average displacement using an ImageJ plugin called track mate.

### Antibacterial Evaluation of the Water Repellent Coating

This experiment involved assessing qualitatively and quantitatively how much bacteria would attach to the coating in comparison to control surfaces. The bacterial adhesion of the two strains of bacteria was assessed using a new method as described below. Bare steel and steel coated with only IPN were chosen as the controls. The experiment was performed in two ways—washed and unwashed adhesion analysis. For the unwashed assay, samples were dunked in a 2.5 cm high liquid column of bacteria suspension for 10 min, and then taken out and stamped on the agar plate. For the washed assay, samples were subjected to an additional step of gentle washing in PBS solution for 5 s before stamping. In each case, the samples were left on the agar plates for 5–10 s. The adhered bacteria on the samples were transferred to the agar plates and the plates were incubated at 37 °C. After 12–16 h of incubation, colonies formed on the agar plates were analyzed to give a visual comparison of the bacterial adhesion on different samples. The residual PBS solution was used to wash the samples were plate‐diluted and the number of colonies in it was enumerated according to the agar plate counting method. A schematic of the procedure is shown in Figure S10 of the Supporting Information. The experiment was repeated in triplicates. Axio observer inverted microscope (ApoTome.2, Carl Zeiss, Germany) under manual focus and with a 5× objective was used to obtain high‐resolution fluorescent images of the substrate imprints on the agar plates. The images were processed using the Zen imaging software (Carl Zeiss, Germany). Since the imprints are quite large, the regions were automatically subdivided into small‐square‐shaped tiles using ZEN. The tiles were then combined via stitching to obtain the whole of the imprint.

### Long‐Term Stability Experiments on the Water Repellent Coating

Here, the water repellent coating to a bacterial suspension was tried to be challenged and the anti‐biofouling effect for a prolonged time while the surface is immersed in it was monitored. The test and the noncoated steel as control samples were exposed to a 2.5 cm column of bacterial suspension of *E. coli* for prolonged durations of time (10 min). The procedure followed was similar to the “washed” assay described in the previous section (Figure S10, Supporting Information). The samples were dunked in the bacterial suspension and incubated at 37 °C for designated periods. Periods chosen were at an interval of an hour starting from 1 and going till 8. After the exposed duration of time, samples were transferred to the PBS solution and given a gentle wash for 5 s, followed by stamping onto the agar plate. The agar plates were incubated for 12–16 h and the PBS solution was plate diluted. To understand the mechanism of plastron collapse, confocal microscopy experiments were performed. For this, a water repellent coating was immersed in a 2.5 cm high column of rhodamine‐B colored milli‐Q water (Figure S6b, Supporting Information). The behavior of plastron subjected to the liquid pressure over time was recorded.

### Synthesis of ZIF‐8

ZIF‐8 in the coating acts as a reservoir of zinc ions that offers bactericidal action. Once the water‐repellency of the coating is compromised, ZIF‐8 in the coating degrades and releases zinc ions that attack the bacteria that come in the vicinity of the coated surface. ZIF‐8 was synthesized via a mechanochemical route as reported elsewhere.^[^ ^35^ ^]^ The reagents zinc acetate (2.08 mmol, Sigma‐Aldrich) and HmIm (16.64 mmol, Sigma‐Aldrich) with a metal‐to‐ligand ratio of 1:8 were placed in a ball milling chamber made of stainless steel, and containing stainless steel balls of 8 mm in diameter. Ball to powder mass ratio was 10:1. Two hours of milling in a Spex 8000 mixer/mill was performed to form a homogeneous paste. After this, deionized water (25 mL) (18 mΩ) was added to the milling chamber and shaken for 15 min, converting the paste into a wet powder. Using filter papers of an average pore size of 200 nm, the powder was vacuum filtered. Unreacted reagents were removed by washing first with deionized water and then with ethanol. The powder was dried at 50 °C overnight in the air.

### Synthesis of FS + ZIF‐8 Suspensions

FS powders were suspended in acetone (analytical grade, Sigma‐Aldrich) at a concentration of 20 mg mL^−1^. In the next step, ZIF‐8 powders were added to the solution to make its weight percent 5%, 10%, 15%, and 20% against FS. The solutions were ultrasonicated for 2 h to finely disperse the particles. These three samples were categorized as test specimens and all the coatings with ZIF‐8 in it have dual‐functional characteristics. But wherever not specified explicitly, dual‐functional coating implies the coating containing 15 wt% ZIF‐8. This is because this 15 wt% ZIF‐8 coating performed the best in bactericidal characterizations as explained in Section 2. The coating fabrication is similar to the water repellent coating except that here 5 mL of FS + ZIF‐8 was sprayed after spraying IPN.

### Surface Characterization of the Dual‐Functional Coating

Dual‐functional coating was characterized by the presence of ZIF‐8 in it using a Bruker system (XRD, D2 Phaser, USA) equipped with copper K*α* radiation with an average wavelength of 1.54059 Å. The EDX elemental mapping was also used to evaluate the distribution. FTIR and contact angle analysis of the dual functional coating was done following the same procedure as for the water‐repellent coating.

### Antibacterial Examination of the Dual‐Functional Coatings

The antibacterial performance of the dual functional coating was evaluated in two conditions where the water repellent coating fails: first was when it was immersed in a contaminated liquid and second when the coating structure was compromised due to physical damage. For the first experiment, the coatings with 5 wt%, 10 wt%, and 15 wt% ZIF‐8 along with bare steel as the control were incubated in a cell culture column of 5 cm height and containing 10^4^–10^5^ cells mL^−1^. It was then transferred to an incubator at 37 °C. After 1 day, 5 days, and 9 days, 1 mL of the culture was plate diluted to see the remaining number of bacterial colonies. The results of contact angle analysis and the aforementioned antibacterial analysis prompted to eliminate all other ZIF‐8 coatings except the 15 wt% one for any further characterizations.

For the second experiment also called the scratch experiment, defects on the surfaces of water repellent coating and the dual functional coating were intentionally created. This experiment was done to evaluate whether the presence of same sized scratches would result in bacterial adhesion on water repellent coating and dual‐functional coatings. The scratches were made toward the center of the sample (indicated with arrows, Figure S14, Supporting Information). A pristine water repellent coating and bare steel were used as reference samples. A 20 µL droplet of bacterial culture in the log phase was anchored at the center. In the case of bare steel due to its hydrophilicity, the droplet spread on the surface (Figure S14a, Supporting Information). In the case of the defective coatings, the droplet remained anchored on the surface (Figure S14b,c, Supporting Information). In the case of pristine water repellent coating, the droplet rolled away due to the small rolling angle of the surface. The droplet was left on the surface for 10 min and then the surface was washed three times with PBS. Each cycle of washing had 1 mL of PBS pipetted onto the surface. The samples were then transferred to 6 well plates. To enable moisture for the bacteria on the surfaces to survive, droplets of PBS were dropped around the samples in the well. Also, adjacent empty wells of the samples were filled with PBS. After this, the well plates were transferred to an incubator at 37 °C. After 24 h of incubation, the samples were taken out and gently washed with PBS three times. Then, the samples were stamped on the agar plates. The agar plates were incubated overnight and then imaged under an Axio Observer microscope. Inductively coupled plasma optical emission spectroscopy instrument (5110, Agilent), and a multielement standard external calibration curve were employed to quantify the release of zinc cations from the immersed coating.

### Fibroblast Cell Culture and Antiviral Experiments

The fibroblast cell line was thawed and cultured in 15 mL of full media including DMEM, 10% fetal bovine serum (FBS), 1× of antibiotic (penicillin, streptomycin, and Neomycin), sodium pyruvate (1 × 10^−3^ m) and supplemented with l‐glutamine (2 × 10^−3^ m) for 3 days in a T75 culture flask. The cells were subcultured at a 1:4 to 1:6 ratio into a T75 culture flask. After trypsinization, cells (8.75 × 10^3^ cells per well) were loaded into 48 well plates with full media overnight, followed by loading virus.

For the antiviral assay, in brief, the coated samples were dipped into the high titre of lentivirus virus (United Bioresearch, Monomeric GFP lentiviral purified lentiviral particles, 10^7^ Tu mL^−1^, 200 µL) for 10 s, then the samples (*n* = 3) dipped into fresh media (named as contaminated surface) for 10 s and underwent cycles of washing (4 times: named as washed 1, washed 2, washed 3, and washed 4) in fresh media. The media from the contaminated surface and subsequent washes were then placed onto fibroblast cells and the transfection was examined. After 24 h, the media was replaced with fresh media in the wells, followed by incubation for another 72 h.

### Cell Fixation and Immunohistochemical Analysis

After removing the media, cells were washed with PBS for 10 min. Paraformaldehyde (4%) was used for fixation for a total of 20 min, followed by three washes with PBS. GFP staining was carried out to enhance the signal from the viruses infected with the GFP‐lentivirus and to calculate the transfection efficiency. GFP primary antibody (1:20 000, donkey anti‐rabbit GFP, Abcam) in PBS with Tween‐20 (0.3%, PBST) and donkey serum (5%, DS) was added to the fixed cells. The solutions were left on the wells overnight at room temperature. The following day, cells were washed three times with PBS and blocked with 10% DS solution in PBST for 30 min. The secondary antibody (1:200, Donkey anti‐rabbit Alexa Fluor 488, Abcam) was applied to cells for 2 h at room temperature. The cells were subsequently washed with PBS, followed by DAPI staining for 10 min. Imaging was observed on fluorescent microscopy (Leica CTR6000) using LAS software to gather three randomly positioned images, and cell numbers were counted by ImageJ software.

### Statistical Analysis

Each experiment including the imaging ones was repeated a minimum of three times wherever not mentioned. All the data are represented as mean ± SD. Analysis of variance (ANOVA) test was performed for statistical significance using the Origin Lab software.

## Conflict of Interest

The authors declare no conflict of interest.

## Supporting information

Supporting Information

Supplemental Movie 1

Supplemental Movie 2

Supplemental Movie 3

Supplemental Movie 4

Supplemental Movie 5

Supplemental Movie 6

## Data Availability

The data that support the findings of this study are available from the corresponding author upon reasonable request.
