# Supplementary material for: Shielding Surfaces from Viruses and Bacteria with a Multiscale Coating
Source: Adv Sci (Weinh). 2022 Jun 3;9(23):2201415. doi: 10.1002/advs.202201415 (PMC9376840; doi:10.1002/advs.202201415)
Supplement: Supplementary file 1 — Supporting Information [file ADVS-9-2201415-s001.pdf]

ADVANCED SCIENCE
Open Access

Supporting Information

for *Adv. Sci.*, DOI 10.1002/advs.202201415

Shielding Surfaces from Viruses and Bacteria with a Multiscale Coating

Deepu Ashok, Mahdiar Taheri, Puneet Garg, Daryl Webb, Pawan Parajuli, Yi Wang, Bronte Funnell, Bradley Taylor, David C. Tscharke, Takuya Tsuzuki, Naresh K. Verma, Antonio Tricoli and David R. Nisbet**

Supporting Information

Shielding surfaces from viruses and bacteria with a multiscale coating

Deepu Ashok, Mahdiar Taheri, Puneet Garg, Daryl Webb, Pawan Parajuli, Yi Wang, Bronte Funnell, Bradley Taylor, David C. Tscharke, Takuya Tsuzuki, Naresh K. Verma, Antonio Tricoli & David R. Nisbet**

D. Ashok, P. Garg, Y. Wang, B. Funnell, B. Taylor, D.R. Nisbet
Laboratory of Advanced Biomaterials, Research School of Chemistry and the John Curtin School of Medical Research, The Australian National University, Canberra, Australia, 2601

D. Ashok, P. Garg, A. Tricoli

Nanotechnology Research Laboratory, Research School of Chemistry, The Australian National University, Canberra, Australia, 2601

M. Taheri, T. Tsuzuki
Laboratory of Advanced Nanomaterials for Sustainability, Research School of Electrical, Energy and Materials Engineering, The Australian National University, Canberra, Australia, 2601

D. Webb

Centre for Advanced Microscopy, Australian National University, Canberra, Australia 2601

P. Parajuli, N.K. Verma

Division of Biomedical Science and Biochemistry, Research School of Biology, The Australian National University, Canberra, Australia, 2601

D.C. Tscharke

John Curtin School of Medical Research, Australian National University, 131 Garran Road, Acton, ACT, 2601, Australia

A. Tricoli

Nanotechnology Research Laboratory, Faculty of Engineering, The University of Sydney, Sydney, Australia, 2006

Email: antonio.tricoli@sydney.edu.au

D.R. Nisbet

The Graeme Clark Institute, Faculty of Engineering and Information Technology & Faculty of Medicine, Dentistry and Health Services, The University of Melbourne, Melbourne, Australia

Email : david.nisbet@unimelb.edu.au

Literature Review

Table S 1 *Previous studies on evaluating bacterial adhesion on superhydrophobic water repellent coatings*

**Reference**	**Coating characteristics**	**Antibacterial Protocol**	**Exposure time**	**% Reduction in adhesion**		**Inference**
				**Gram-positive**	**Gram-negative**	
Monofunctional/repellent surfaces						
**Hizal et al.**[1]	Nanopillared Aluminium modified with Teflon	Parallel plate flow chamber.<br>- For the static assay: The substrates were held inside the chamber with the bacterial suspension.<br>-Dynamic assay: The bacterial suspension flown through the chamber at a certain rate	1 h	99.9 % for dynamic assay,<br>98 % for static assay	99.4 % for dynamic assay,<br>99 % for static assay	The decrease in bacterial adhesion is attributed to the high roughness of the 3D pillars, that provided minimized contact area for bacteria

**Crick et al.**[2]	A highly rough thin film of Silicon elastomer	Immersion in the bacterial suspension	1 h	80%	58%	The static assay was performed and the coated surface had a significant reduction in adhered bacteria
**Privett et al.**[3]	Xerogel coating of nanostructured fluorinated silica colloids, fluoroalkyl silane, silane backbone	A parallel plate flow cell -Dynamic assay: The bacterial suspension flown through the chamber at a certain rate	1.5 h	99 %	98.2%	The highly rough fluorinated surface provided reduced contact area for the bacteria
**Bartlet et al.**[4]	Titania nanotube arrays modified with (heptadecafluoro- 1,1,2,2-tetradecyl) trichlorosilane	Substrates in a well plate was treated with 1 mL of bacterial suspension	6 h & 24 h			Reduced adhesion after 24 h and no biofilm formation observed
**Fadeeva et al.**[5]	Nanorough titanium fabricated by femtosecond laser ablation	Incubation of the surface with a bacterial droplet on it	18 h	-	-	Selective antifouling against a gram-negative strain
**Dual-**						

functional surfaces						
Agrawal et al.[6]	Cotton fabrics cotton with ZnO nanoparticles anchored by aminopropyl triethoxy silane	Incubation of the surface in bacterial suspension and serial dilution to estimate the reduction in bacteria	24 h	98%	98%	Demonstrated a multifunctional coating resistant to abrasion, UV irradiation, ultrasonic and chemical washing along with good antibacterial properties.
Wu et al[7]	Sequential deposition of branched poly(ethylenimine), silver NPs and fluorinated decyl polyhedral oligomeric silsesquioxane on fabrics	The coated fabrics were immersed in a bacterial culture, followed by optical density measurements up to 16 hours	16 h	-	-	The test samples with silver NPs recorded almost no increase in optical density over time, suggesting the ions can bind with bacteria and annihilate them
Gao et al.[8]	Cotton fabrics were first	Zone of inhibition assay	24 h	-	-	Multifunctional coatings on cotton

	coated with silver nanoparticles using polydopamine as adhesive and then with polydimethylsiloxane or polyimide with hydrophobic properties.					fabrics with anticorrosion, self cleaning, chemical stability and antibacterial properties
Liu et al.[9]	Hierarchical micro-nano structures of polyelectrolyte coated with fluoro-silane and silver NPs embedded	Incubating the samples in culture followed by imaging	1 & 7 days	-	-	Superhydrophobicity prevented the adhesion upto 7 days and then silver ions were released killing the cells
Tripathy et al. [10]	Hierarchical polydimethylsiloxane (PDMS) surface embedded with copper hydroxide NPs	Incubating the samples in the culture followed by plate dilution	0, 3, 18 and 24 h	-	-	After 18 h, no cells found on the test surface against nearly $5log_{10}$ observed on control

Zhang et al.[11]	Hierarchical bimetallic composite surface with metal oxide coating on porous copper foam substrate and silver ions embedded	- Incubation in the culture followed by optical density measurement.<br>- Zone of inhibition analysis	-	-	Combination of bimetallic composition, presence of metal oxides and large surface area endows an enhanced antibacterial effect in wet and dry conditions

Materials and Methods

Synthesis of IPN

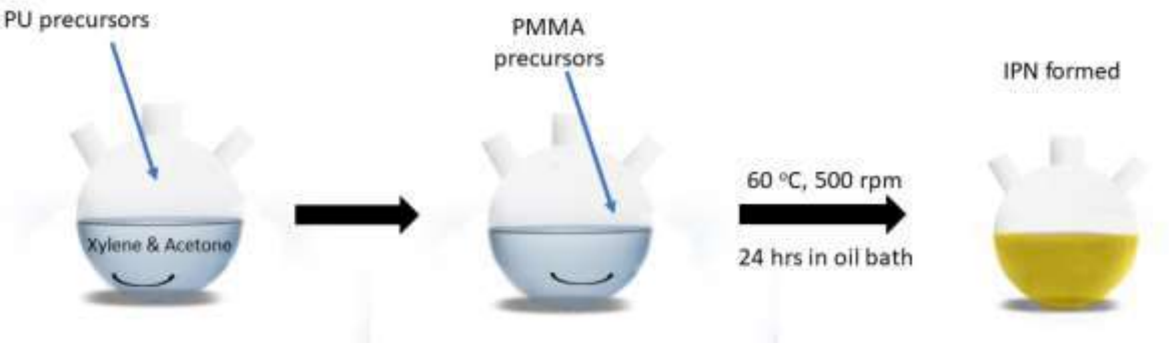

Figure S1: *Schematic showing the synthesis of IPN*

Preparation of FS

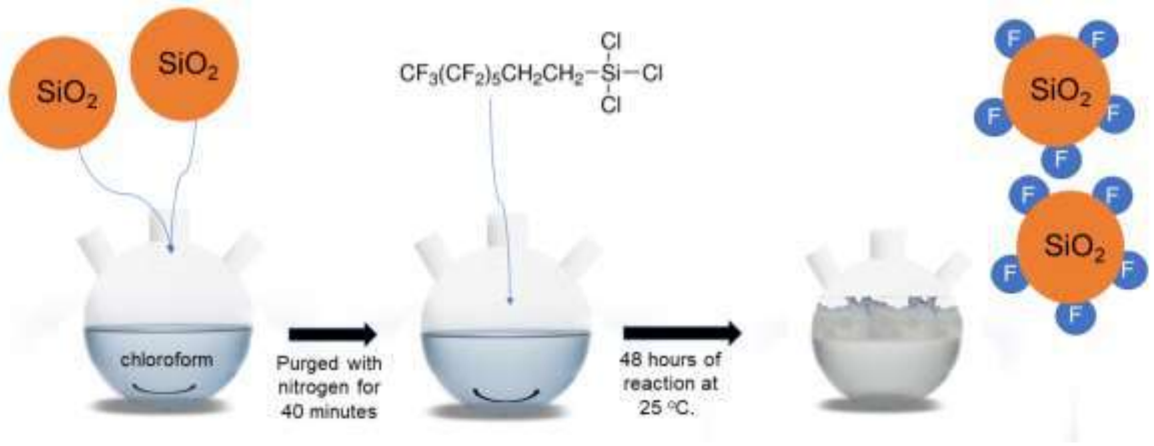

Figure S2: *Schematic showing the preparation of FS*

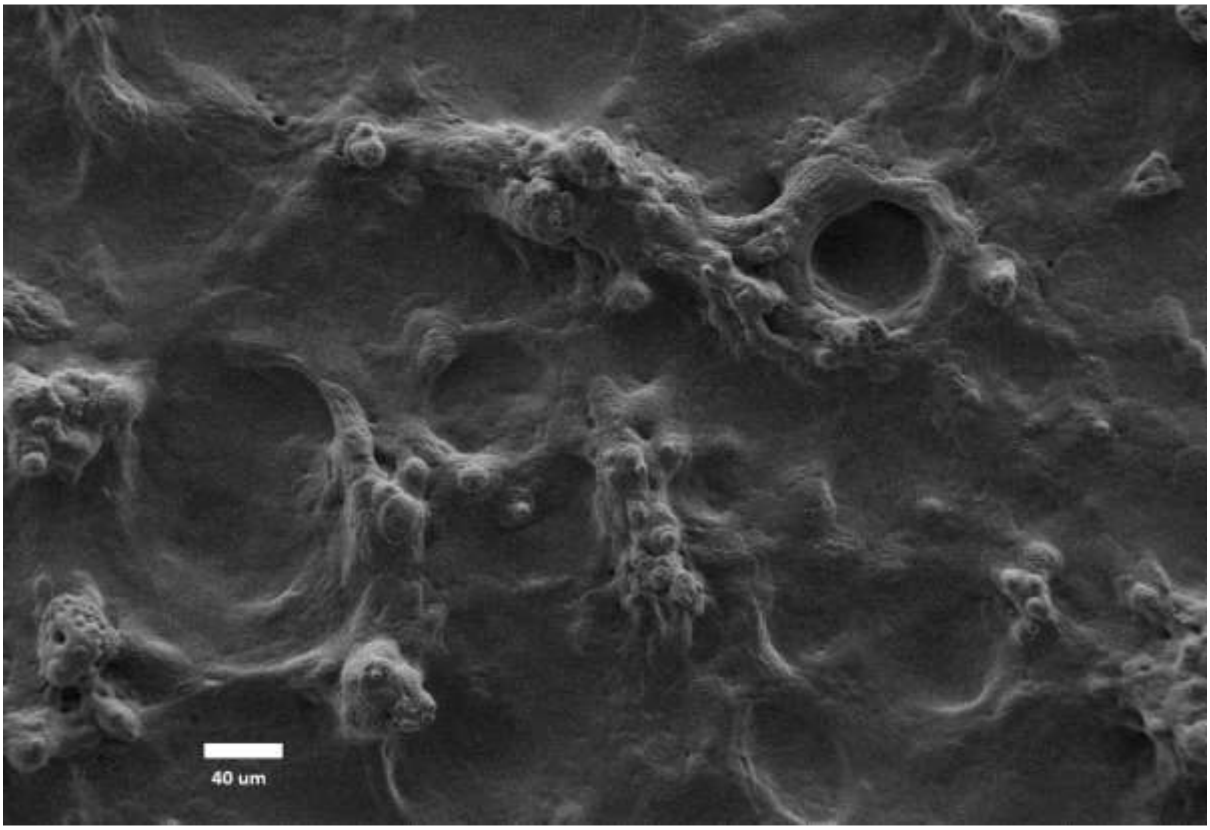

Figure S3: *SEM micrograph of the microstructured IPN layer*

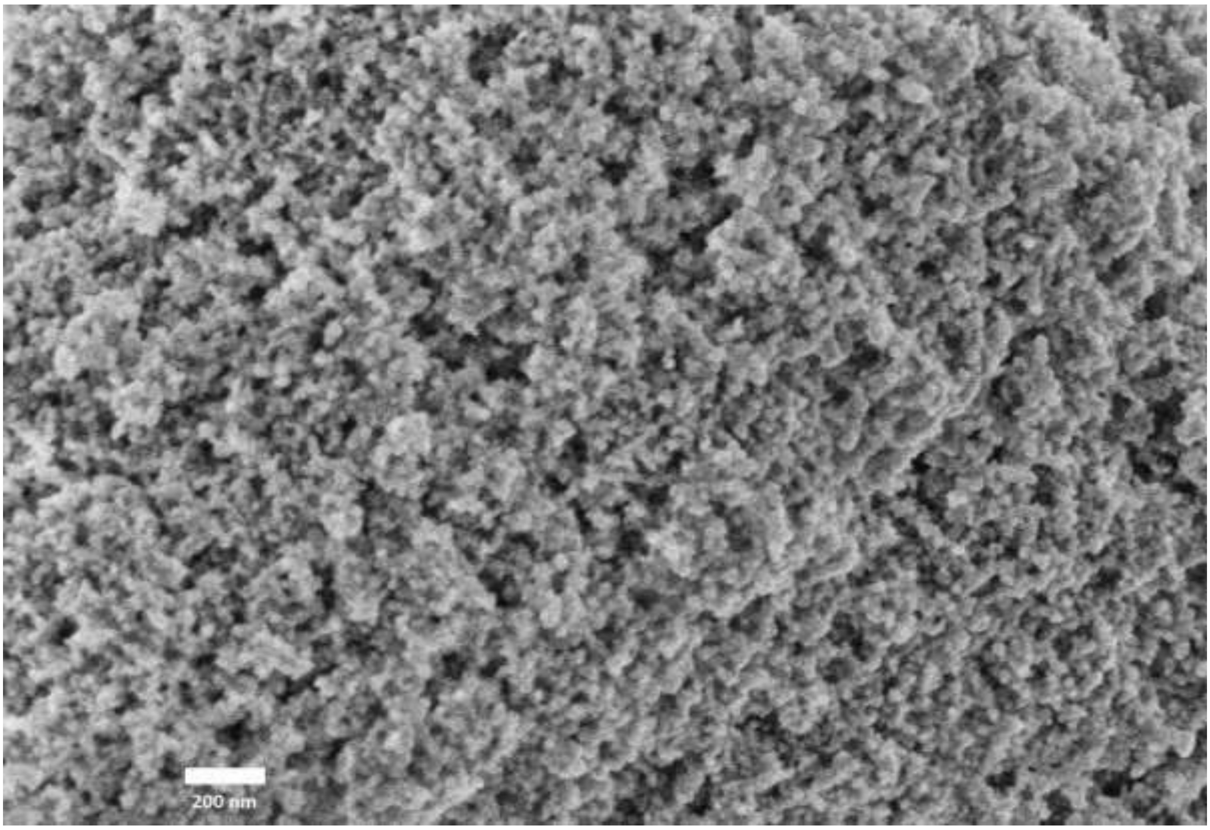

Figure S4: *SEM micrograph of the nanostructured FS layer*

FS

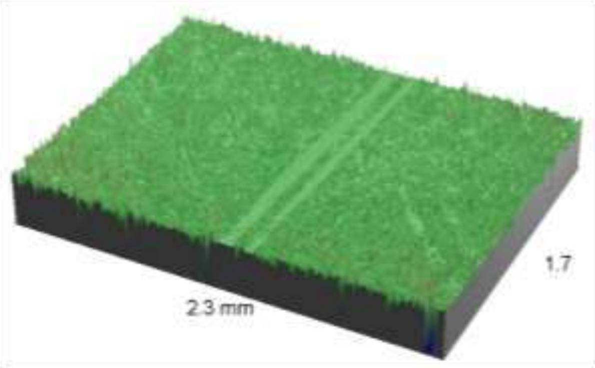

***Figure S5**: White Light Interferometry color maps of the IPN+FS layer showing the roughness of the coating. IPN+FS layer recorded a roughness value of 165.93 ± 63.12 nm. A scratch was imparted in the middle of the coating as seen to measure the thickness of the coating. The thickness came out to be 2.67 ± 0.42 microns (n=3).*

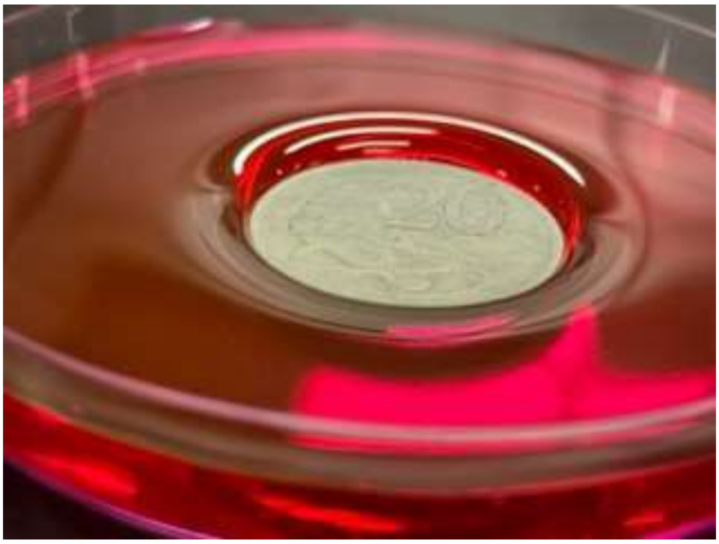

***Figure S6:** Demonstration of the lotus leaf effect and the uniformity of our coating applied on a textured surface (a 20 cent Australian dollar coin) immersed in rhodamine coloured water.*

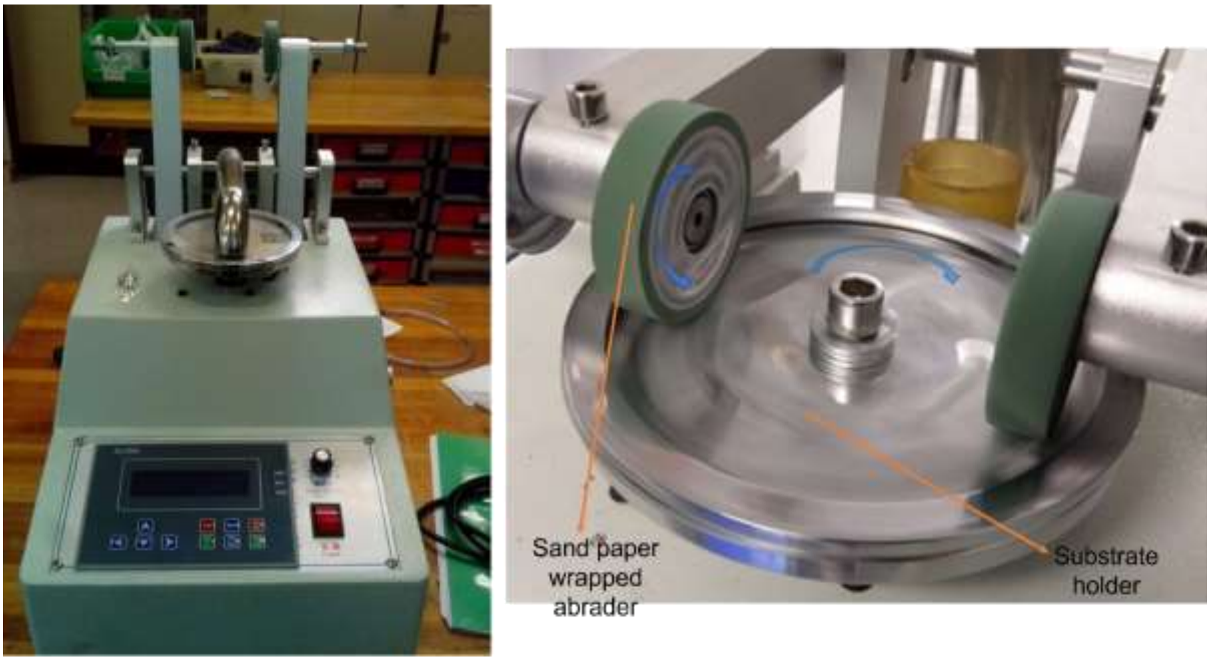

Figure S7: *Photograph showing the instrument used for testing abrasion resistance of our coating. The analysis subjects the specimens to scratching due to the presence of sand paper on the abrader.*

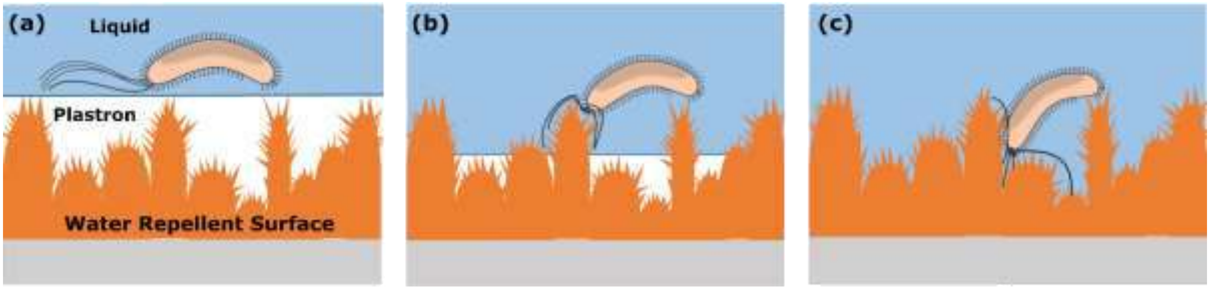

Figure S8: *Surface shielding mechanism. (a-c) Schematic representation of the surface shielding mechanism initially preventing the adhesion of bacteria to the coating.*

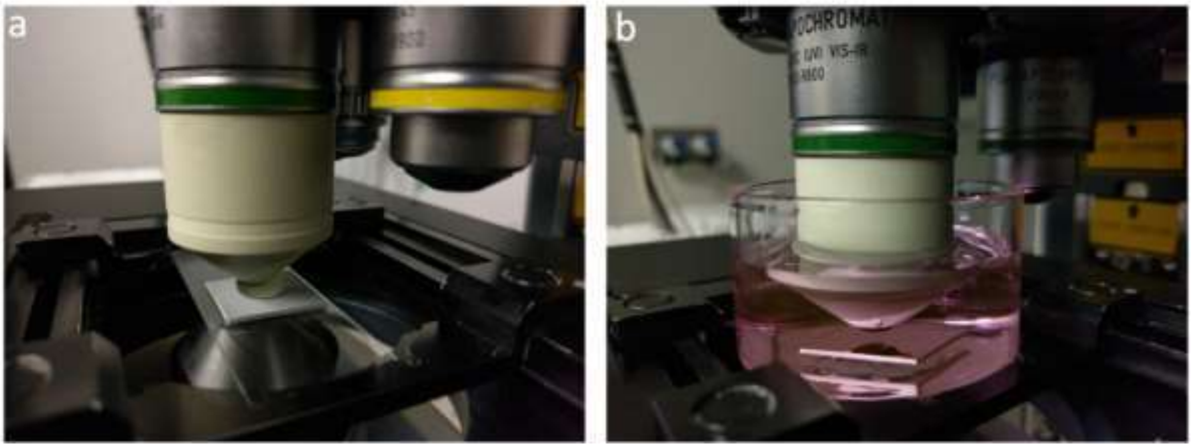

Figure S9: *Mechanistic studies using CLSM. (a) Anchoring a droplet of bacterial suspension on top of the substrate. (b) Long exposure studies by immersing a water repellent coating in a dye-coloured column of water and recording the event of plastron burst.*

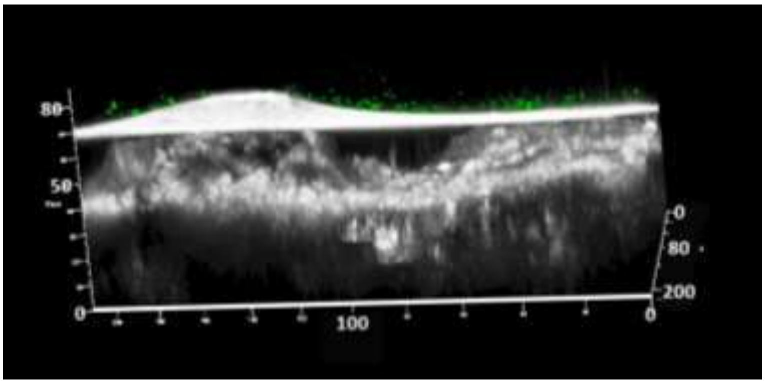

Figure S10: *CLSM image showing a different orientation of the coated substrate with bacteria lying on the plastron. The silvery curved layer represents the liquid meniscus, and the green represents bacteria. Scale is in microns.*

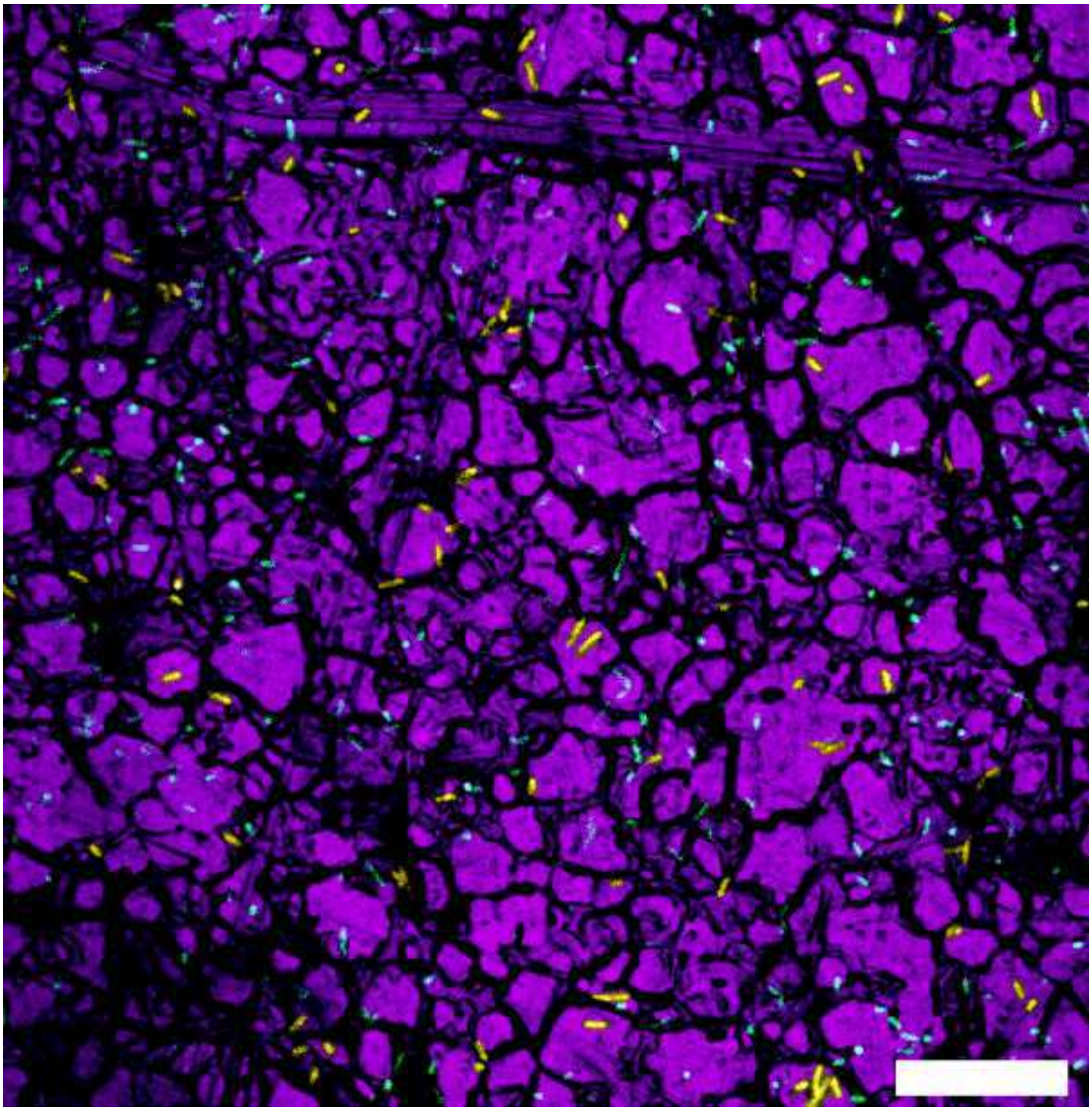

Figure S11: *Dynamic response of bacteria on non-coated steel surface at 1 minute. Attached bacteria are false coloured yellow. Scale is 25 microns.*

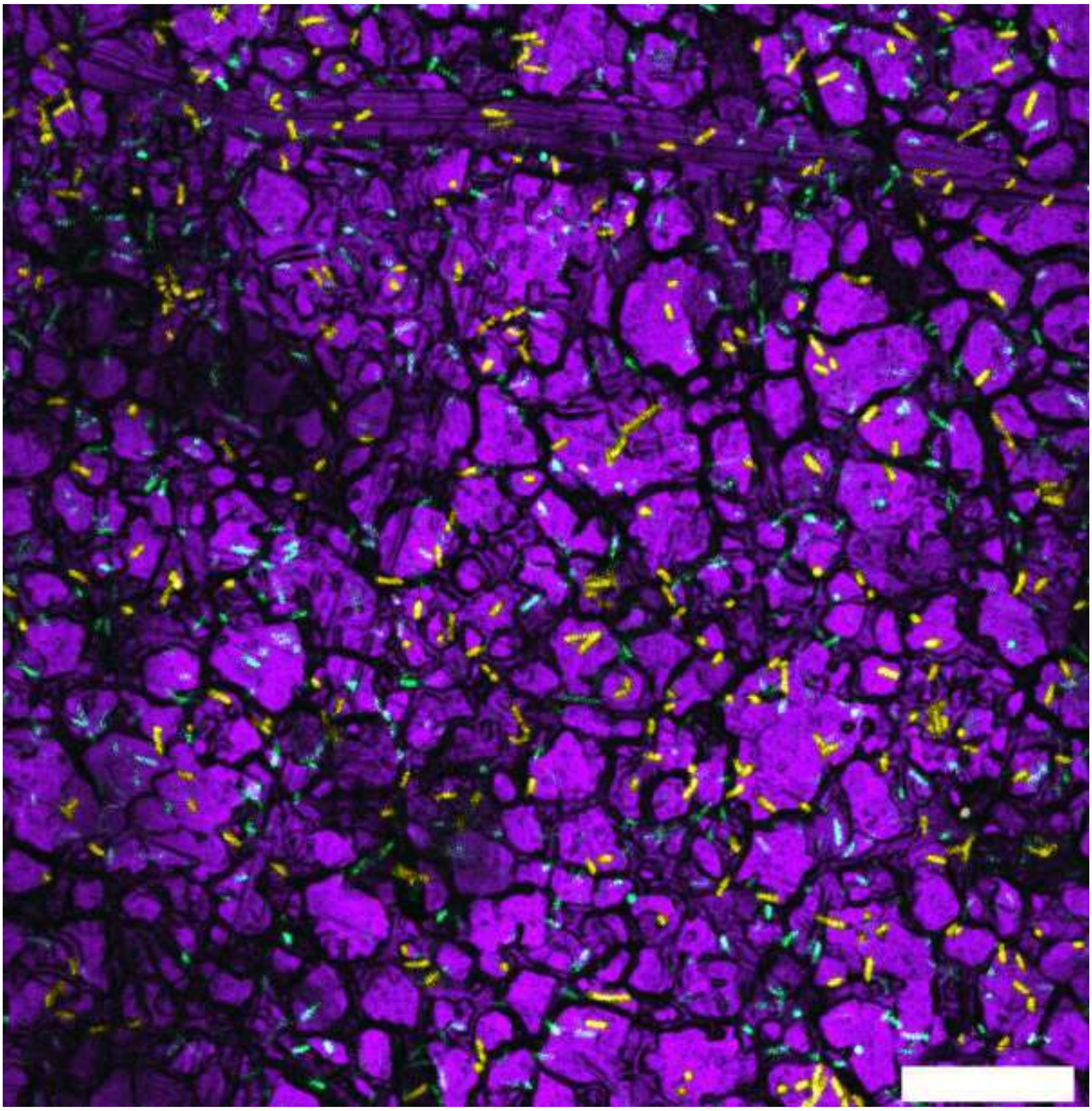

***Figure S12**: Dynamic response of bacteria on non-coated steel surface at 8 minutes. Attached bacteria are false coloured yellow. Scale is 25 microns.*

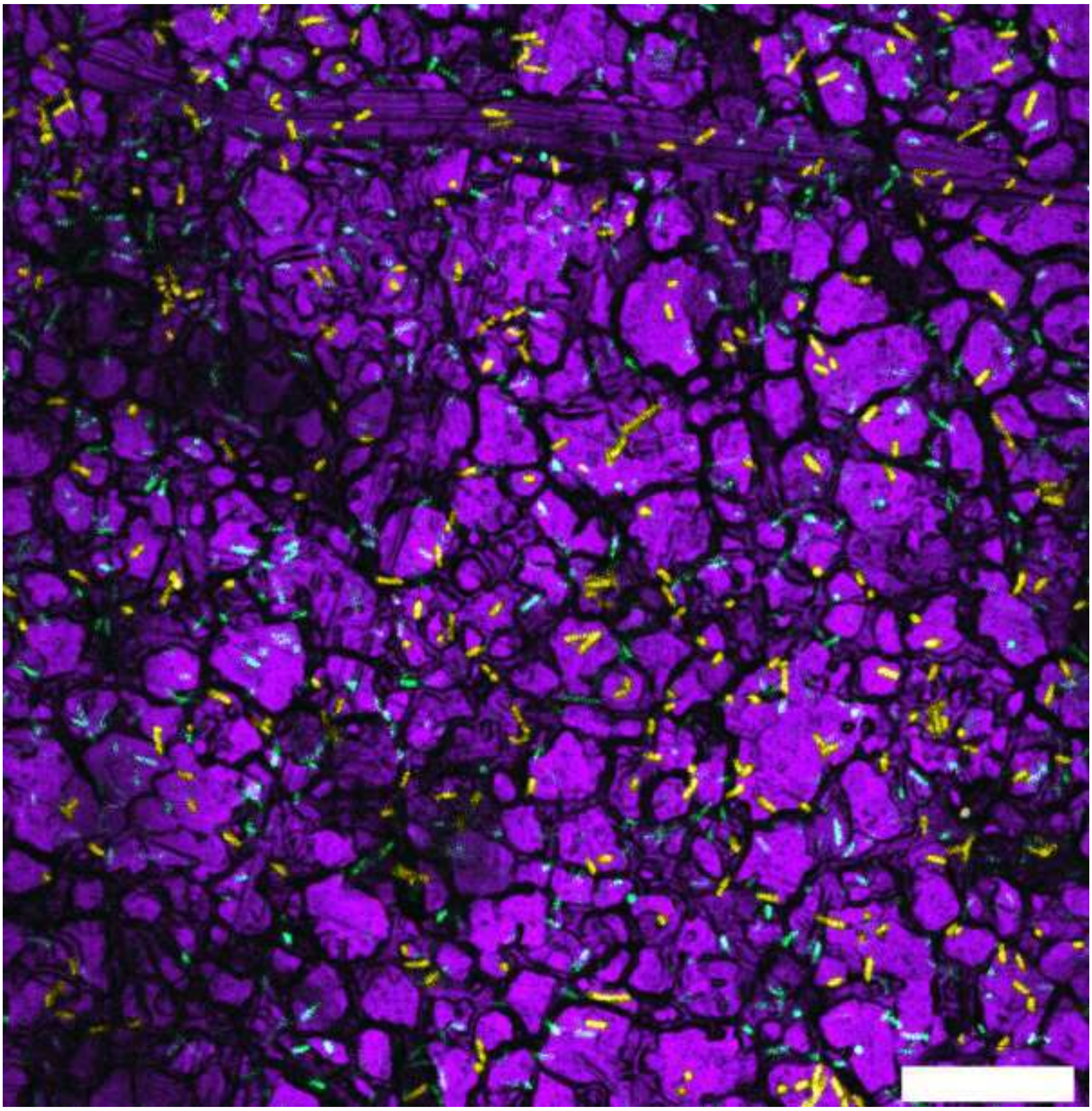

***Figure S13**: Dynamic response of bacteria on non-coated steel surface at 14 minutes. Attached bacteria are false coloured yellow. Scale is 25 microns.*

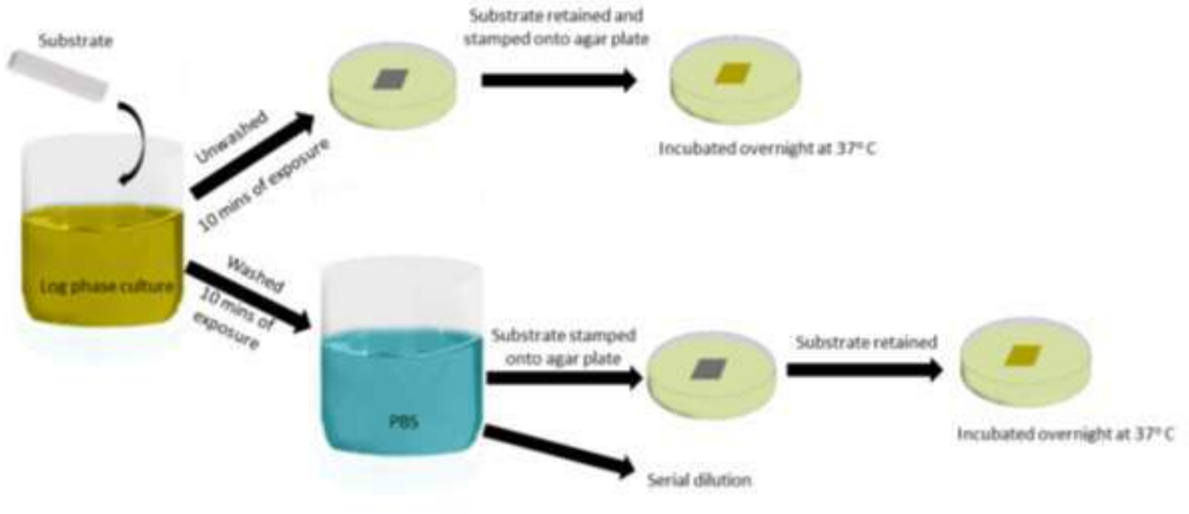

Figure S14: *Schematic representation of evaluating the quantitative and qualitative adhesion of bacteria*

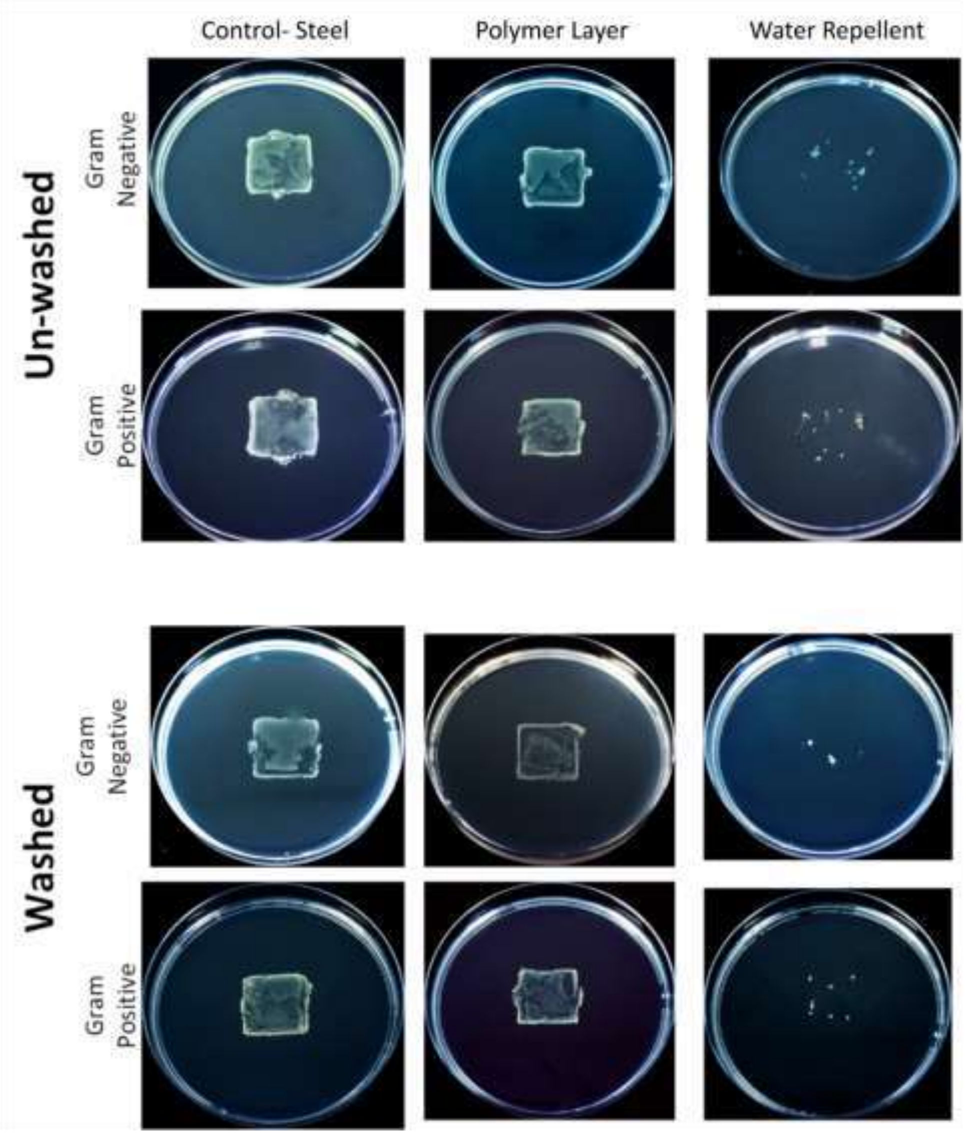

Figure S15: *Photographs of the agar plates stamped with specimens that were exposed to bacteria.*

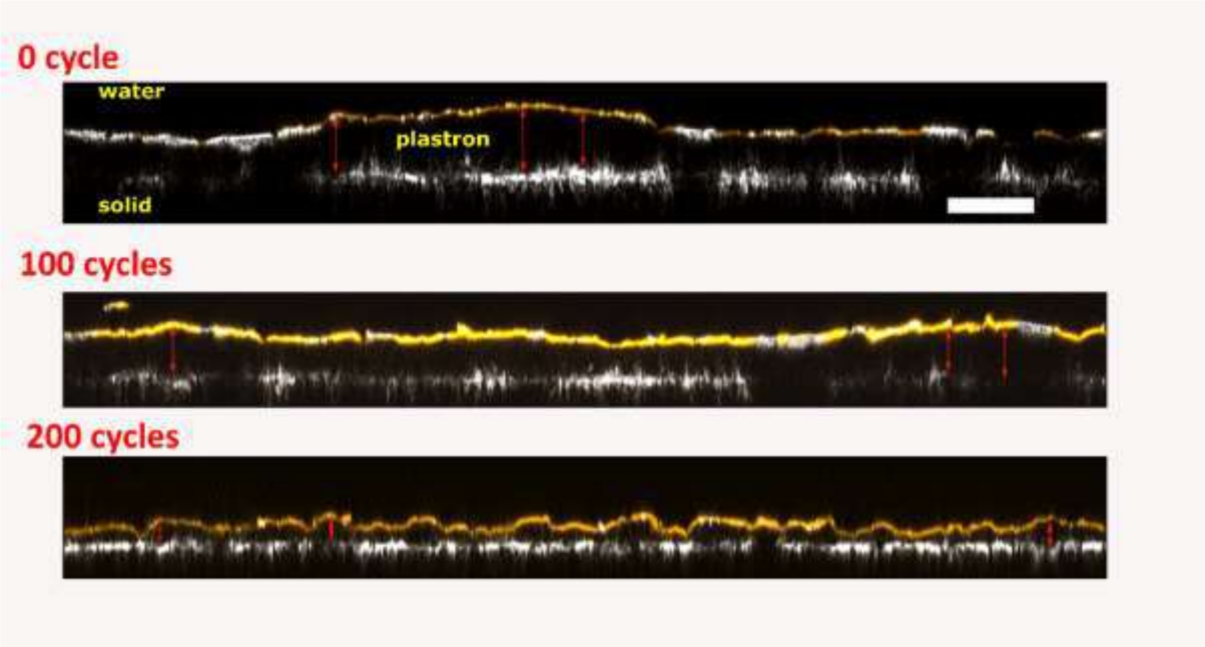

Figure S16: *CLSM orthogonal projections of un-abraded coating (0 cycles) versus the same coating abraded to 100 and 200 cycles. Scale bar is 50 µm. The plastron thicknesses were measured at the highest points, indicated by red arrows. For un-abraded sample, the thicknesses were 33, 33.3 and 38.4 µm. For 100 cycles, it is 27.8, 25.5 and 29.4 µm. For 200 cycles, the values are 13.5, 12.6, 24 µm. The samples at these cycles were chosen because of the significant decrease in plastron thickness that is apparent.*

Dual functional coating

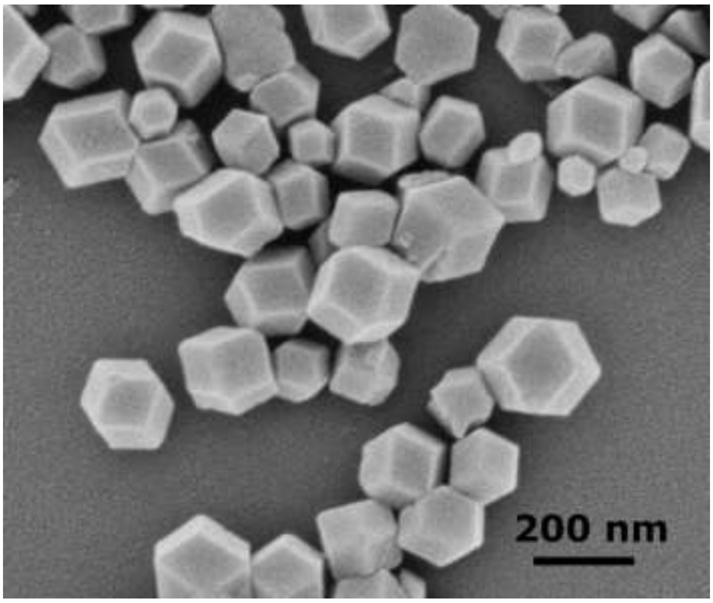

Fig S17: *(a) SEM image of the ZIF-8 powders*

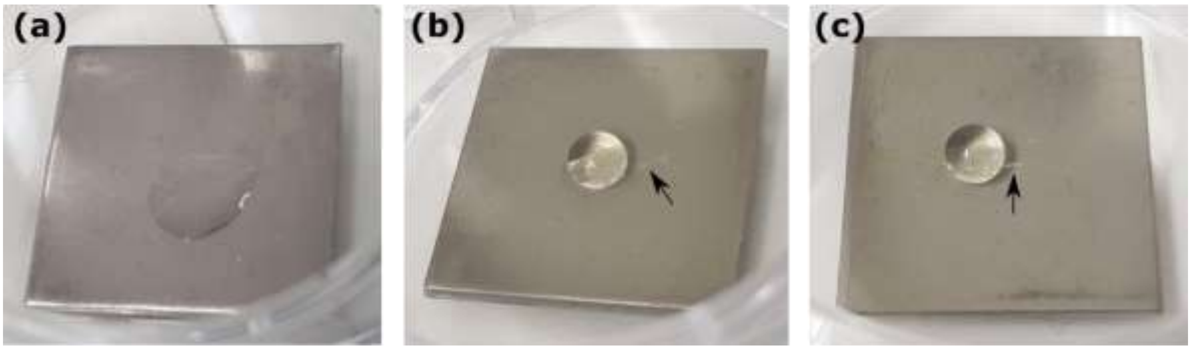

Figure S18: *Photographs of bacterial droplet behaviour on different samples; (a) bare stainless steel as the control, (b) scratched water repellent coatingcoating, and (c) scratched water repellent coating coating containing 15 wt% of ZIF-8*

Table S 2 *Contact angle and rolling angle values of the coatings as a function the content of antimicrobial ZIF-8.*

ZIF-8 wt%	Contact angle (degrees)	Rolling angle (degrees)
**0 (water-repellent)**	157 ± 0.47	0
**5**	161 ± 4.67^{o}	0
**10**	158 ± 1.41^{o}	0
**15 (dual-functional)**	155 ± 0.66^{o}	0
**20**	149 ± 1.63^{o}	8 ± 1.72

.

Note 1: Failure kinetic of the water repellent shielding mechanism

We conducted an in-operando optical investigation of the surface protection mechanism of our water repellent coating structure, determining potential failure and bacterial adhesion mechanisms (**Figure S5**).

The immersion depth is an important parameter often overlooked in plastron-stability studies (**Table S1**).[12] Following extended submersion of the coating, the liquid pressure on the plastron layer causes its gradual dissolution. As the plastron continues to degrade and thin, it exposes parts of the underlying coating surface to the bacteria allowing for their adhesion. We have observed that the multi-scale roughness of our coating structure increases the stability of the plastron layer up to 4 h. This is significantly longer than previous studies on water repellent coatings reporting plastron loss within 1 to 1.5 h (**Table S1**).

For exposures of 4 to 6 h, a rapidly increasing number of bacteria colonies is observed (**Figure 6c-e**). While longer time results in complete colonization of the bacteria-challenged area (**Figure 6f-h**). This trend was quantified by serial dilution data showing that up to 4 h a significant reduction of the number of bacteria colonies is observed with respect to the bare steel surface (**Figure 6f-h**, **Table S3**). Notably, the water repellent coatings were able to decrease the number of bacteria colonies by 99.8% for up to 4 h continuous immersion. This indicates a plastron layer stability in the range of 3-4 h.

Upon 5-6 h continuous immersion, the bare and coated steel samples resulted in similar bacteria colony densities (**Table S3**). Notably, for immersion times of 7 h and longer, the water repellent coatings displayed an increasingly larger number of bacteria colonies (**Table S3**). This is attributed to the micro and meso-scale roughness of the water repellent coatings that provide a larger area for bacteria adhesion upon dissolution of the plastron layer. Previous studies on the use of water repellent coatings to prevent bacteria adhesion have not been able to achieve comparable reductions in bacteria colonies for such an extended period of immersion time (**Table S1**). Significant reduction in bacteria colonies was reported only for significantly shorter immersion times of 1 h or less. One of the most recent studies that

analysed the plastron collapse of water repellent coatings observed less than 90% reduction for 1 h.[12]

Table S3: *Number of bacteria colonies after incubation with bacteria for 1hour to 8 hours*

**Incubation time with bacteria (h)**	**Number of bacteria colonies of Steel (CFUs/mL)**	**Number of bacteria colonies of Water Repellent (CFUs/mL)**
1	$2.6\times10^{5} \pm 4.5\times10^{4}$	144 ± 57
2	$1.1\times10^{6} \pm 4.2\times10^{5}$	13266 ± 2819
3	$1.4\times10^{7} \pm 1.7\times10^{6}$	20144 ± 2723
4	$2.6\times10^{7} \pm 3.0\times10^{6}$	$8.4\times10^{5} \pm 2.4\times10^{5}$
5	$3.2\times10^{7} \pm 9.8\times10^{6}$	$1.7\times10^{7} \pm 1.6\times10^{6}$
6	$1.4\pm10^{8} \pm 3.7\times10^{7}$	$1.0\times10^{8} \pm 4.5\times10^{7}$
7	$2.6\times10^{8} \pm 1.0\times10^{8}$	$8.1\times10^{8} \pm 3.4\times10^{8}$
8	$1.3\times10^{9} \pm 5.3\times10^{9}$	$1.6\times10^{10} \pm 4.3\times10^{9}$

Stability of the plastron layer and influence of multiscale roughness.

It has been reported that the disintegration time of the plastron layer depends from the immersion depth as an increase in the liquid pressure results in an exponential increase in the diffusion of air into the liquid. [13] Similar correlations may be determined as a function of the temperature.[14] Here, we have observed that the lifetime of the plastron layer is also a function of the surface roughness, and thus in the case of hierarchical surfaces with multi-scale roughness, the plastron layer lifetime depends on the local topography of the coating. For this reason, the transition from non-wetting to wetting regimes does not occur abruptly and at the same time for the entire area of the coating.

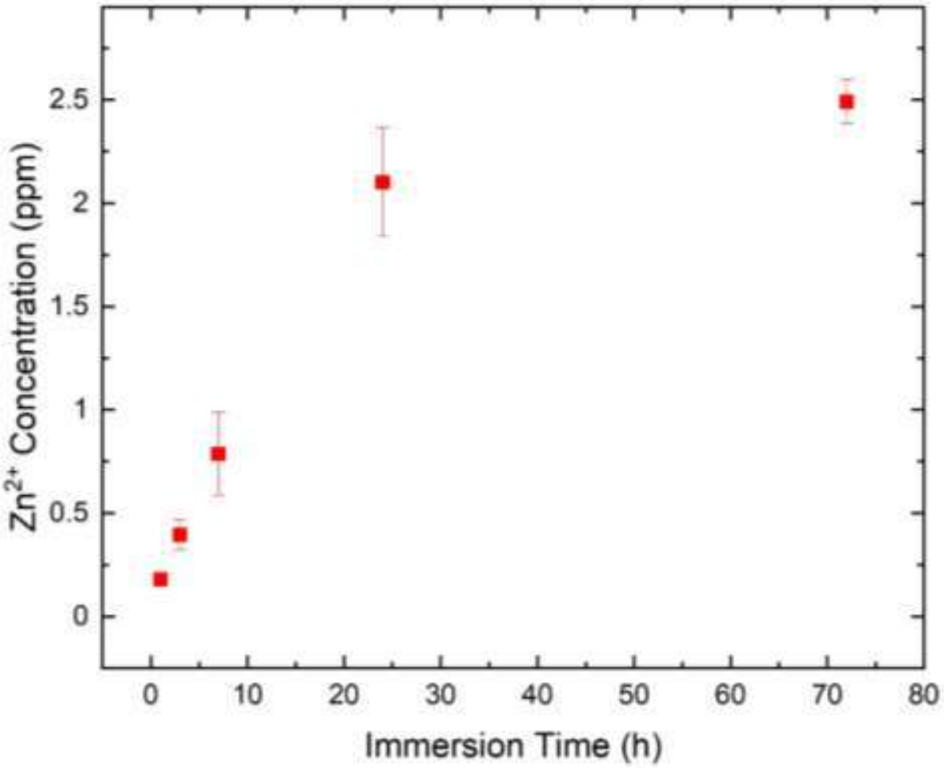

Figure S19: *Plot showing the release of Zn ions from the dual functional coating immersed in deionised water (n=3). The trend is in agreement with **Figure 6** where there is complete loss of plastron by around 8 hours. The increase in Zn ion can be observed at this point.*

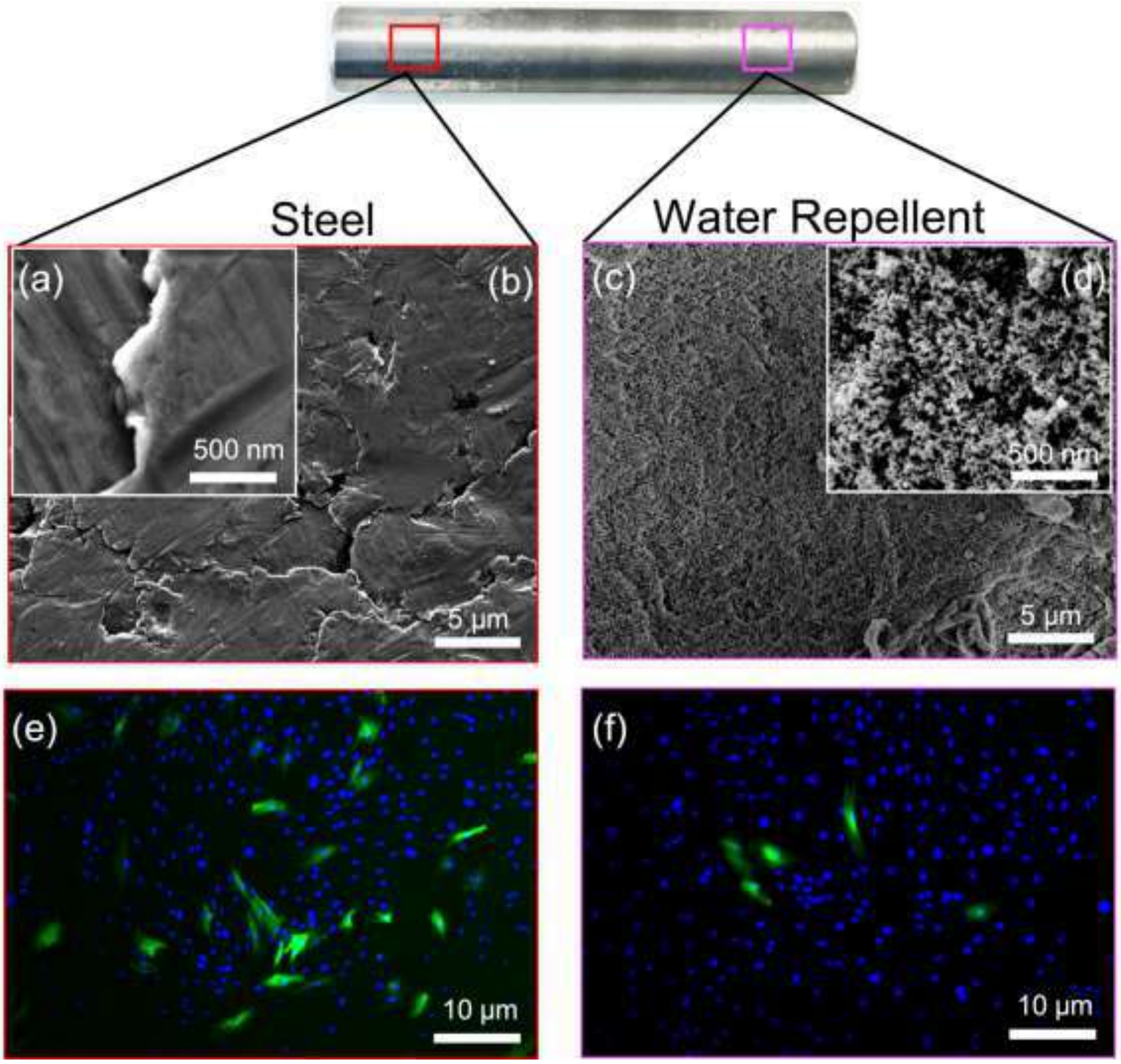

Figure S20: *SEM images of steel rods (uncoated steel (a, b) and coated surface (water repellent, c,d)) at different magnification and representative images of cell transfection for contaminated surface (e, f)*

References

[1] F. Hizal, N. Rungraeng, J. Lee, S. Jun, H. J. Busscher, H. C. van der Mei, C.-H. Choi, ACS Applied Materials & Interfaces 2017, 9, 12118.

[2] C. R. Crick, S. Ismail, J. Pratten, I. P. Parkin, Thin Solid Films 2011, 519, 3722.

[3] B. J. Privett, J. Youn, S. A. Hong, J. Lee, J. Han, J. H. Shin, M. H. Schoenfisch, Langmuir 2011, 27, 9597.

[4] K. Bartlet, S. Movafaghi, L. P. Dasi, A. K. Kota, K. C. Popat, Colloids and Surfaces B: Biointerfaces 2018, 166, 179.

[5] E. Fadeeva, V. K. Truong, M. Stiesch, B. N. Chichkov, R. J. Crawford, J. Wang, E. P. Ivanova, Langmuir 2011, 27, 3012.

[6] N. Agrawal, J. S. J. Tan, P. S. Low, E. W. M. Fong, Y. Lai, Z. Chen, Advanced Materials Interfaces 2019, 6, 1900032.

[7] M. Wu, B. Ma, T. Pan, S. Chen, J. Sun, Advanced Functional Materials 2016, 26, 569.

[8] Y.-N. Gao, Y. Wang, T.-N. Yue, Y.-X. Weng, M. Wang, Journal of Colloid and Interface Science 2021, 582, 112.

[9] T. Liu, B. Yin, T. He, N. Guo, L. Dong, Y. Yin, ACS Applied Materials & Interfaces 2012, 4, 4683.

[10] A. Tripathy, A. Kumar, S. Sreedharan, G. Muralidharan, A. Pramanik, D. Nandi, P. Sen, ACS Biomaterials Science & Engineering 2018, 4, 2213.

[11] M. Zhang, P. Wang, H. Sun, Z. Wang, ACS Applied Materials & Interfaces 2014, 6, 22108.

[12] G. B. Hwang, K. Page, A. Patir, S. P. Nair, E. Allan, I. P. Parkin, ACS Nano 2018, 12, 6050.

[13] R. Poetes, K. Holtzmann, K. Franze, U. Steiner, Physical Review Letters 2010, 105, 166104.

[14] A. Martínez-Gómez, S. López, T. García, R. de Francisco, P. Tiemblo, N. García, ACS Omega 2017, 2, 8928.
